# Mediterranean and Northern Iberian gene pools of wild *Castanea sativa* Mill. are two differentiated ecotypes originated under natural divergent selection

**DOI:** 10.1371/journal.pone.0211315

**Published:** 2019-02-12

**Authors:** B. Míguez-Soto, J. Fernández-Cruz, J. Fernández-López

**Affiliations:** Department of Forest Breeding, Forestry Research Centre of Lourizán, Pontevedra, Spain; University of Melbourne, AUSTRALIA

## Abstract

Nine wild Iberian provenances of *Castanea sativa* Mill. grouped in two gene pools, North Iberian Peninsula and Mediterranean, were evaluated for several adaptive traits in two provenance–progeny trials with the aim of evaluating the role of natural selection in shaping adaptive variation and increasing our understanding of the genetic structure of this species, as well as reporting complete information on the genetic variation among and within the studied populations. An annual growth rhythm experiment was evaluated during the first 3 years after establishment for phenology, growth, stem form and survival, and a periodic drought-stress experiment was evaluated for dry weight, growth, survival and other related drought traits in both well-watered and drought-stress treatments. The high genetic variability reported in both trials is largely due to the genetic variation among populations. The significant differences reported between quantitative genetic and neutral marker differentiation indicated the local adaptation of these populations through directional selection, mainly for phenology, growth and biomass allocation. A clinal variation among populations was determined through correlations of phenology with latitude and xerothermic index of the provenances, showing that central and southern Mediterranean populations had earlier phenology than northern populations and that drought played a relevant role in this differentiation. The significant correlation between phenological traits and the ancestry values in the Mediterranean gene pool supported the different pattern of behavior between both gene pools and also indicated the existence of two ecotypes: xeric and mesophytic ecotypes, corresponding to Mediterranean and North Iberian gene pools, respectively. The results obtained in the drought-stress experiment confirmed that, in general terms, xeric populations showed a greater adaptability to drought, with more developed root systems and higher survival than northern populations. Moreover, the genetic variability observed within populations indicated the potential response capacity of Iberian *C*. *sativa* populations to undergo fast adaptive evolution.

## Introduction

Genetic diversity provides the fundamental basis for evolution by natural selection [1] and, therefore, its preservation within and among populations of a species is necessary to safeguard its potential to adapt to future environmental changes [2]. Two types of genetic diversity should be considered: neutral genetic variation, which includes stochastic processes such as gene flow and genetic drift, and adaptive genetic variation mainly determined by selection [3– 4]. The balance of these evolutionary forces determines the level of differentiation among populations, enhanced by divergent selection and genetic drift and constrained by gene flow. On the other hand, the genetic variation within populations has gene flow as a main force maintaining the genetic variability and stabilizing natural selection and genetic drift acting as constraint processes [5, 6].

The presence of genetic variation among and within populations of temperate broadleaved and conifer species for the adaptively important traits evaluated in provenance tests has been widely reported [7–10]. The broad distribution of many of these species, and consequently the different environmental conditions under which the trees grow, usually imply a high level of variability in various phenotypic traits that is expected to reflect underlying genotypic variations necessary for adaptation to local environmental conditions. In some temperate broadleaved species of scattered distributions and limited gene flow, such as *Acer platanoides* and *Fraxinus excelsior*, most genetic variation for traits related to growth rhythm is observed among populations [11–13]. On the contrary, greater genetic variability is observed within, rather than among, populations of species with more continuous distributions and effective pollen and seed dispersal mechanisms, such as *Betula pendula* [13] and *Alnus glutinosa* [11]. Temperate forest phenology usually reflects clinal (longitudinal, latitudinal and altitudinal) variation patterns among populations of broadly distributed temperate forest species because of the effects of different climatic factors along their geographical distribution [8, 10, 14], mainly a combination between winter chilling, photoperiod and temperature [15]. For example, some observations in common provenance tests of temperate broadleaved trees were that southern populations of *Fagus sylvatica* [16] and *Quercus petraea* [17] flushed earlier than northern populations, while northern populations of *B*. *pendula* [18] and *Populus tremula* [19] showed an earlier bud set in comparison with southern populations. The main phenological changes registered in European forests in the last decades were a clear reaction to temperature increment of the global warming [20]. The advanced phenology observed in response to earlier and warmer springs increased the risk of spring frost damage [21] due to greater temperature fluctuations also caused by climatic change. Consequently, phenology and other fitness-related traits such as tolerance to drought and heat are especially relevant in studies on genetic diversity and tree responses to climatic change [22].

Different molecular markers have been widely used for neutral genetic diversity studies of temperate forest species in Europe [23]. The general observation is that forest tree species showed high levels of genetic diversity within populations and low among populations [24, 25], contrary to the differentiation across populations observed in quantitative traits associated with local adaptation. Despite the development of different methods in the last years related to genome wide research trying to associate genetic and adaptive variations, molecular markers cannot fully capture the complexity of the polygenic adaptive traits [26]. In this sense, the technique based on comparisons between the coefficient of differentiation among populations at a neutral marker locus (*F*_*ST*_) [27] and the analog coefficient of differentiation for a quantitative trait (*Q*_*ST*_) [28, 29] remain useful tools for evaluating the actions of natural selection that affect adaptive traits [30], under the assumption that populations are at drift–migration equilibrium and that the mutation rate is negligible in comparison with the other evolutionary forces [31]. The non-significant differences between both parameters suggest that a trait under additive genetic control is neutral with respect to selection [32] and, consequently, it is assumed that differentiation observed at genes underlying this quantitative trait is mainly caused by drift. Local adaptation through directional natural selection is suggested when the coefficient of quantitative differentiation is sufficiently greater than the differentiation at neutral markers, whereas the contrary case is interpreted as stabilizing selection [28, 31, 33].

*Castanea sativa* Mill. (sweet chestnut) is a broadleaved species with a long-range scattered distribution across the Mediterranean region of Europe and western Asia. This region covers 2.5 million ha in Europe [34, 35], of which 256.380 ha are in Spain (IV Spanish National Forest Inventory). At the northern margin of its distribution, development of *C*. *sativa* is limited by low temperatures, which imply frost damage and reduced seed production, and, at the southern margin, its survival is limited by drought [36]. In its distribution area, this species is present as high-forest, simple coppices, coppices with standards and grafted orchards. The widespread use of grafted varieties could have had a strong impact on the genetic structure of wild populations [37, 38].

With respect to the genetic and geographical structure of *C*. *sativa*, defined through several studies using different genetic markers, the general conclusion is that populations of this species are distributed in three main clusters: eastern and central Turkey, western Turkey and Greece, and a European cluster from Italy and the Iberian Peninsula [39, 40]. In the Iberian Peninsula, populations have been divided into Western Mediterranean and Northern Iberian Peninsula gene pools, with the latter subdivided into Cantabrian and Atlantic Galician groups including mainly coastal populations [38, 41, 42]. The Northern Iberian groups could be explained by several refugia formed in this area during the Quaternary [43]. Moreover, a recent paleoecological study of a *C*. *sativa* population in central Iberian Peninsula provided the first evidence of the existence of a refuge located in this area [44]. The Western Mediterranean gene pool, also identified on the Italian Peninsula and traditionally related to the fruit cultivation of *C*. *sativa* [35, 37, 40, 45], is also present in the northwest of Iberian Peninsula, in the inner mountains of Galicia, probably introduced through the naturalization of grafted varieties [38].

Because of the distribution of *C*. *sativa* and its restricted dispersal of pollen and seeds, an important genetic differentiation is expected among populations because of local environmental adaptations, although other processes such as phenotypic plasticity [46] and domestication [47] could have affected this adaptive differentiation. Several provenance studies based on the genetic variation of adaptive traits related mainly to phenology and growth, reported important differences among natural European and Iberian populations of *C*. *sativa* [48–52]. Specifically, provenance trials conducted in Iberian Peninsula indicated a latitudinal clinal variation for several adaptive traits; northern populations showed later flushing and bud set, more vigorous growth, higher stem straightness and higher resistance to frost than central and southern Spanish populations [49, 51, 52] This north-south variation has been related to drought and frost as relevant factors in the natural selection that shaped the actual structure of *C*. *sativa* populations. Moreover, in terms of drought-stress, different ecophysiological responses were observed in European populations of *C*. *sativa* depending on the origin sites’ conditions [53–57]. All these patterns of geographical variation identified in these adaptive traits seem to indicate, in general terms, a natural origin of the Iberian chestnut populations. In relation to the genetic variation within natural populations, fewer studies have been carried out but the general observation is that among-population differences were greater than within population differences [51, 52].

Despite the important information reported recently on the geographic structures of *C*. *sativa* populations, there are no studies relating the gene pools identified with genetic markers to the clinal variation in adaptive traits observed in this species. There are also very few specific studies, and no references using natural stands in Iberian Peninsula, about adaptive response of *C*. *sativa* to drought, despite the demonstrated importance of drought as a selective force [58] and the consideration of *C*. *sativa* as a drought-sensitive species [59].

Based on the existing knowledge of wild Iberian chestnut populations, we hypothesized that both gene pools identified in the Iberian Peninsula with neutral markers, Mediterranean and North, correspond to two different ecotypes adapted to their climatic conditions as a consequence of natural selection caused by drought in the central and southern populations and by cold temperatures in the north of Iberian Peninsula. In order to demonstrate this hypothesis, an adaptive genetic variation study was conducted in two provenance-progeny trials for nine wild populations, previously genotyped with 9 microsatellites (SSRs), representing the geographic distribution of *C*. *sativa* in the Iberian Peninsula with the objectives of: 1) to evaluate the role of natural selection in the differentiation of the populations of both gene pools; 2) to increase our understanding of the geographic structure of the Iberian populations of *C*. *sativa* through a joint analysis relating genetic parameters estimated for several adaptive traits to the ancestry of populations and to geographic and climate data of their origin sites; 3) to describe both groups of populations according to their adaptability to drought-stress; 4) to evaluate their potential for an evolutionary adaptive response to environmental changes.

To achieve the proposed objectives, the following analysis were performed:: 1) quantitative genetic variation among and within wild *C*. *sativa* Iberian populations and estimate of genetic parameters through mixed model methodology for several adaptive traits related to annual growth rhythm and drought-stress in both nursery and greenhouse provenance-progeny experiments; 2) comparisons between *Q*_*ST*_ and *F*_*ST*_ for the evaluated traits through simulation and resampling methods, and 3) correlations of ancestry, estimated with SSRs, geographic and climatic data with phenotypic observations.

## Materials and methods

This study did not involve endangered or protected species. No specific permissions were required for the collection of plant material in the stands described below because they do not belong to protected or restricted areas. The owners of the lands gave their word of mouth to conduct the material collection on these sites. Moreover, all the experimental trials of this study were developed in the Forest Research Center of Lourizán, institution that provided approval for the authors to conduct the experiments and to which the corresponding author belongs. The collection of material and the experimental trials were carried out within the framework of the project “092CastaneaREG (2004–2006)” in compliance with legality.

### Plant material

**Fig 1:**
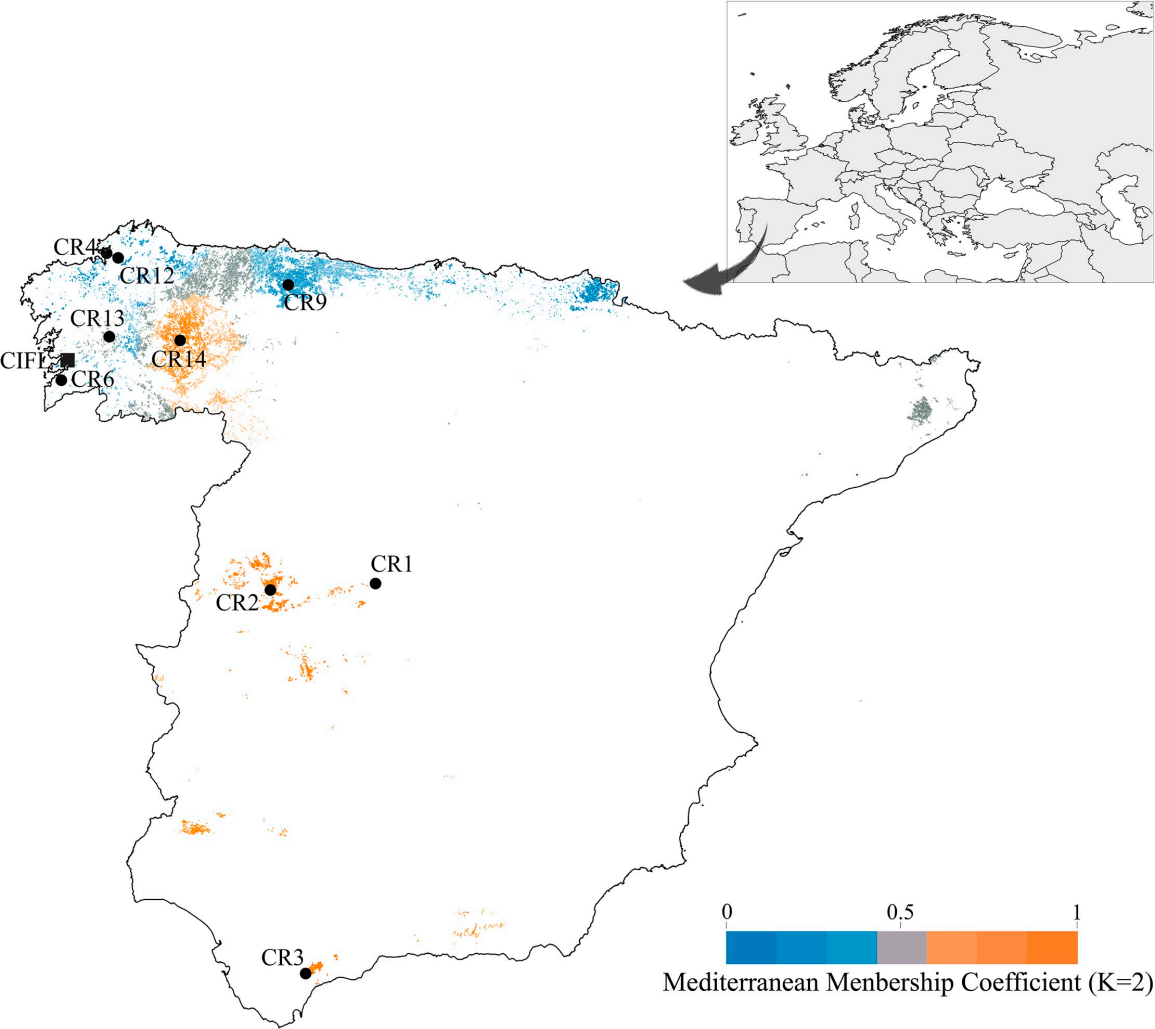
Geographical distribution of the nine *C*. *sativa* stands studied and the location of the FRCL nursery. The distribution area of the species (Fourth Spanish National Forest Inventory) is shown with two color gradients, which represent the ancestry membership proportions assuming two ancestral populations (K = 2), estimated by Fernández-Cruz and Fernández-López (2016) for 29 naturalized *C*. *sativa* stands distributed through Spain and Europe. The values range from 0 (Northern Iberian Peninsula gene pool) to 1 (Mediterranean gene pool). Blue areas belong to the Northern Iberian Peninsula gene pool, while orange areas are related to the Mediterranean gene pool. The most intense colors represent the highest ancestry values of each gene pool. The areas of admixed populations located in the Northwest of the Iberian Peninsula are represented by grey colors. An inverse distance weighting interpolation was performed with Quantum GIS software to define the extension of each genetic cluster.

Nine wild *C*. *sativa* stands located in nine geographically distant sites throughout the Iberian chestnut distribution area (Fig 1) were examined in this study. The genetic structure of these nine populations, along with other 20 wild Iberian and European populations of *C*. *sativa*, were previously analyzed by Fernández-Cruz and Fernández-López [38] using STRUCTURE software to define the most probable number of clusters and to calculate the membership of each individual in each cluster. According to the ancestry values of the Mediterranean cluster, two main gene pools were identified: the Northern Iberian Peninsula gene pool, containing the northern and northwestern coastal stands of San Cibrán (CR6), Maniños (CR4) and Eume (CR12), with membership ancestry coefficients lower than 0.1 (q < 0.1), as well as Nandiello (CR9, q = 0.17), and the Mediterranean gene pool, containing the central and southern stands of El Tiemblo (CR1, q = 0.82), Hervás (CR2, q = 0.86) and Ronda (CR3, q > 0.9). The northern stand of Catasós (CR13) is an admixed population (q = 0.53) between these two gene pools that originated by the naturalization of cultivated varieties of Mediterranean origin. Mercurín (CR14) is a northern stand belonging to the Mediterranean cluster (q = 0.78) of probable introduced origin, located in an area where chestnut orchards are dominant.

The sampled stands grow under different climates depending on their locations (Table 1). The northwestern stands of CR6, CR4 and CR12 grow in the Atlantic area, with mild temperatures, a humid climate and a very low annual temperature oscillation. CR13 and CR14 stands also grow in a cold humid climate but with greater annual temperature oscillations than the previously mentioned stands, being stand CR14 subjected to some summer drought conditions. The northern stand of CR9 grows in intermediate conditions between the two previous groups. The CR1 stand grows in the high elevations of the mountains of Central Spain, with the coldest climate among the selected locations and with a very high annual temperature oscillation. Finally, the stands of CR2 and CR3 grow in the Central West and South West of Spain, respectively and have a mild mean annual temperature and dry summer. The CR2 stand grows, moreover, in a high temperature oscillation.

**Table 1:** Geographic origin and climatic conditions for the *C*. *sativa* spanish populations, and the number of progenies and individuals included in the annual growth and drought-stress experiments.

Location^a^						Climate^b^						Trials^c^	
Code	Population	Region	Longitude	Latitude	Alt (m)	P (mm)	T (°C)	MTD (°C)	ATD (°C)	SP (mm)	X_i_	AG	DS
CR1	El Tiemblo	Ávila	04° 31' 57" W	40° 20' 43" N	1200	1196	9.1	31.14	42.1	228	0	28 (434)	10 (400)
CR2	Hervás	Cáceres	05° 51' 52" W	40° 15' 13" N	940	1266	13.4	29.56	41.54	109	26.5	24 (238)	10 (370)
CR3	Ronda	Málaga	05° 18' 04" W	36° 32' 14 "N	700	1238	14.1	23.86	28.28	67	68.4	14 (74)	10 (198)
CR4	Maniños	A Coruña	08° 11' 41" W	43° 26' 39" N	90	1338	12.6	17.4	31	124	0	19 (45)	10 (160)
CR6	San Cibrán	Pontevedra	08° 40' 56" W	42° 11' 17" N	225	1881	13.8	19.1	32.56	149	0.22	17 (56)	10 (114)
CR9	Nandiello	Asturias	05° 45' 51" W	43° 13' 04" N	400	938	12.5	22.46	35.73	169	0	17 (96)	10 (172)
CR12	Eume	A Coruña	08° 02' 16" W	43° 24' 17" N	214	1987	15.5	20.84	33.67	263	0	23 (168)	10 (148)
CR13	Catasós	Pontevedra	08° 05' 32" W	42° 38' 12" N	580	1799	10.9	28.01	40.08	221	0	26 (429)	10 (298)
CR14	Mercurín	Lugo	07° 09' 51" W	42° 38' 18" N	650	1297	10.4	25.42	40.14	145	4.04	24 (238)	10 (380)

^a^Locational data: Alt, altitude

^b^Climatic data: P, annual precipitation; T, mean annual temperature; MTD, difference between the mean temperature of the warmest and coldest months; ATD, difference between the absolute maximum and minimum temperatures; SP, summer precipitation; Xi, xerothermic index [Xi = Σ(2Tm-Pm) if Pm < 2 Tm, or Xi = 0 if Pm > 2 Tm, where Tm is the monthly mean of the maximum and minimum temperatures (°C) and Pm is the monthly precipitation (mm)]

^c^Trial data: AG, annual growth experiment; DS, drought-stress experiment. Number of families and total number of individuals (in parentheses) per population

In the experiments, each stand was represented by the open-pollinated progenies of 30 selected trees. Seeds of each tree were collected during the autumn of 2004, and the seed lots were conserved in a refrigerator at 3°C until 15 March 2005, when the seeds were directly sown in 2 l pots with a substrate composed of peat, chipped pine bark and perlite in the same volume proportions in the Forestry Research Centre of Lourizán (FRCL) greenhouse. The germinated seedlings were grown at the same temperature and received normal watering. In July 2005, plants were split between two experiments to evaluate the annual growth rhythms and periodic drought-stress responses of Iberian *C*. *sativa* populations under extreme and intermediate conditions.

### Experimental designs and drought treatment

The annual growth rhythm experiment contained 1,778 seedlings distributed in 192 families (Table 1), which were established outdoors in the FRCL nursery. The trial was planned as a randomized block design with 20 blocks and one-tree plots, although there was a certain unbalance in the number of families and individuals within families because of the poor conservation of some seed lots. The 2,240 plants, belonging to 90 families, included in the drought trial (Table 1) were separated equally into two different greenhouse chambers, one for the well-watered treatment and one for the periodic drought-stress treatment. The trial design was a randomized single-tree plot with one seedling per family in each of the 10 blocks per treatment. The number of seedlings used was 2,240, and there was some unbalanced data collected for several families.

Plants destined for the drought trial were watered to full field capacity by flood irrigation for 15 min every day until the drought treatment was started on 28 July 2005. Seedlings of the well-watered treatment were maintained at full field capacity and a moderate temperature, whereas plants of the stress treatment were subjected to a high temperature and periodic drought cycles. These cycles consisted of maintaining the seedlings without irrigation until the substrate saturation decreased to approximately 40% of the weight at full capacity and then re-watering to full field capacity (S1A Fig). There were 21 drought cycles during the experiment, and the substrate saturation level was determined from the weight of the pots every day, with 30 pots in the treatment chamber and 10 pots in the control chamber being randomly selected from different points of each chamber. During the 10-week duration of the experiment, the average indoor air temperature and relative humidity were, respectively, 21 ± 4°C and 80 ± 12% for the well-watered treatment, designated as T21W, and 27 ± 3°C and 51 ± 9% for the periodic drought-stress treatment, designated as T27D (S1B Fig).

### Assessments

In the annual growth rhythm experiment, terminal and lateral flushing (TF and LF) were evaluated in the spring of 2007 and 2008 at the beginning of the second and third growing season, respectively. Flushing was recorded using the eight-point scale modified from Solignat and Chapa [60] by Fernández-López et al. [49]: 1 (dormant buds), 2 (initial swelling of buds evident), 3 (green leaves shorter than brown scales), 4 (green scales longer than brown scales), 5 (leaf nerves evident), 6 (shoot length < 5 cm), 7 (shoot length 5–10 cm) and 8 (shoot length > 10 cm). For LF, we considered the predominant stage of lateral bud burst. Seven measurements were performed for this trait during the months of March and April, and the analyzed variable was the number of days since January 1 until the stage in which buds were considered flushed (score 3). Bud set (BS) was assessed at the end of the growing season in the same years, 2007 and 2008, and it was evaluated with the following four-stage scale [50]: 1 (plant in active growth), 2 (terminal buds initiated but still very small, the shoot less herbaceous, and stipules of younger leaves still green), 3 (terminal bud developed but not of definitive size, with most stipules having dropped or, if still attached, yellow or brown) and 4 (buds fully developed on the completely lignified shoot, stipules usually dropped, and leaves of a coriaceous consistency). Assessments of BS were performed every 7 days during June and August in the annual growth rhythm experiment and every 10 days during the first month of the periodic drought-stress experiment. We recorded this variable as the number of days since January 1 until the stage in which buds were considered set (score 4). Height (H) was measured in three consecutive years (2006, 2007 and 2008), and root collar diameter (RCD) was measured in 2007 and 2008. Both growth traits were measured in centimeters. Straightness (STR) was evaluated in 2008 according to the following three-point STR score: 1, straight; 2, bent; and 3, sinuous. For apical dominance (AD), also evaluated in 2008, a four-point scale was used to designate the number of branches competing with the main leader: 1, no competing branches; 2, one competing branch; 3, two or more competing branches; and 4, no erect shoot at the top of the tree. Survival (S), which was assessed in 2006, 2007 and 2008, was measured as a binary variable, by recording the value of 0 (dead) or 1 (alive).

For the periodic drought-stress experiment, the number of days for the initiation of seed germination was recorded. Height was evaluated in July at the beginning of the application of the drought-stress treatment and in the last week of August, after BS. RCD, defoliation (DEF), number of axillary buds sprouting and number of secondary branches (NSB) were measured after six weeks of drought initiation, at the end of the experiment. DEF was evaluated using the following five-point scale: 0, full green canopy; 2, some yellowing and up to 25% DEF; 2, 25%–50% DEF; 3, 50–75% DEF; and 4, 75–100% DEF. BS and dried apex (DA) were measured from the beginning of the trial until the end of the growing season. We recoded DA as a binary variable with the values 1 (green) and 0 (dry). Finally, after the 10 weeks of treatment, S was recorded and seedlings were harvested to assess dry weight of stems (SDW), roots (RDW) and leaves (LDW) and to estimate the total dry weight (TDW) as the sum of the three separate dry weights. Dry weight traits were measured in grams, and for their assessment, the three plant parts were separated and put in paper bags to be dried to constant weights in an oven for 24 h at 80°C.

### Statistical analyses for quantitative traits

All statistical tests were carried out using the SAS 9.4 statistical package [61]. Statistical analyses were performed using the MIXED procedure for continuous variables and the GLIMMIX procedure for binary and categorical traits. GLIMMIX procedure allows to fit generalized linear mixed models for non-normal data through link functions. In our study, the best fit to experimental data was provided by the binomial distribution and logic link function for binary traits and the multinomial (ordered) distribution and cumulative logic link function for categorical traits. Variance components were estimated through restricted maximum likelihood and Laplace (maximum likelihood approximation) methods in the MIXED and GLIMMIX procedures, respectively. Both statistical procedures also allow the estimation of the best linear unbiased estimate of the fixed effects and the best linear unbiased prediction (BLUP) of the random effects.

Two linear models were used for the annual growth rhythm experiment, one for the analysis of each population separately and the other for the joint analysis across populations. Both models included the family random effect to estimate additive variances and derived genetic parameters within and among populations. The population random effect in the second model allows estimating, in addition, *Q*_*ST*_ values among populations. The final fitted models included the fixed effect of block and, in order to increase the goodness of fit, the second model was simplified by removing the random effect of the interaction between population and block, which was never significant. The models were set as follows:

$$X_{ikl}=\mu +B_{i}+F_{k}+e_{ikl}$$ (1)

for the analyses of each population separately, and

$$X_{ijkl}=\mu +B_{i}+P_{j}+F_{k\left(j\right)}+e_{ijkl}$$ (2)

for the analyses across all populations, where *X*_*ikl*_ and *X*_*ijkl*_ indicate the values of single observations, *B*_*i*_ indicates the fixed effect of the *i*^th^ block, *P*_*j*_ indicates the random effect of the *j*^th^ population ~*N*(0, $\sigma_{p}^{2}$), *F*_*k*_ and *F*_*k*(*j*)_ indicate, respectively, the random effect of the *k*^th^ family in Eq (1) and the random effect of the *k*^th^ family within the *j*^th^ population in Eq (2) ~*N*(0, $\sigma_{f}^{2}$), and *e*_*ikl*_ and *e*_*ijkl*_ indicate the random error terms ~*N*(0, $\sigma_{e}^{2}$).

In the statistical two models were used for the periodic drought-stress experiment, the number of days for the initiation of germination and H before the experiment started was used as covariates, fixed effects in the model statement of the MIXED or GLIMMIX procedures, to diminish the influence of some confounding maternal effects. For comparative purposes, the first model for the analyses across all populations for each treatment separately was fitted following the same criteria as the annual growth rhythm experiment. The second model for the analyses across treatments included the factor of treatment and the effects of its interaction with population and family. Treatment and population were included as fixed factors in the final fitted model. Both models were simplified by removing the effect of the interaction between population and block, which were never significant. The linear models for the periodic drought-stress experiment were:

$$X_{ijkm}=\mu +DG_{m}+H_{m}+B_{i}+P_{j}+F_{k\left(j\right)}+e_{ijkm}$$ (3)

for the analyses of each treatment separately, and

$$X_{ijklm}=\mu +DG_{m}+H_{m}+T_{l}+B_{i\left(l\right)}+P_{j}+F_{k\left(j\right)}+TP_{lj}+TF_{lk\left(j\right)}+e_{ijklm}$$ (4)

for the analyses across the two treatments, where *X*_*ijkm*_ and *X*_*ijklm*_ indicate the individual observations, *μ* indicates the mean population, *DG*_*m*_ and *H*_*m*_ indicate, respectively, the fixed effects of days of germination and the H of the *m*^th^ individual, *T*_*l*_ indicates the fixed effect of the *l*^th^ treatment, *B*_*i*_ and *B*_*i*(*l*)_ indicate, respectively, the fixed effects of the *i*^th^ block and the fixed effect of the *i*^th^ block within the *l*^th^ treatment, *P*_*j*_ indicates the random effect 2 ~*N*(0, $\sigma_{p}^{2}$) and the fixed effect of the *j*^th^ population in Models 2 and 3, respectively, *F*_*k*(*i*)_ indicates the random effect of the *k*^th^ family within the *j*^th^ population ~*N*(0, $\sigma_{f}^{2}$), *TP*_*lj*_ indicates the fixed effect of the interaction between the *l*^th^ treatment and the *j*^th^ population ~*N*(0, $\sigma_{pt}^{2}$), *TF*_*lj*_ indicates the random effect of the interaction between the *l*^th^ treatment and the *k*^th^ family within the *j*^th^ population ~*N*(0, $\sigma_{pt}^{2}$), and *e*_*ijkm*_ and *e*_*ijklm*_ are the residual random errors ~*N*(0, $\sigma_{e}^{2}$).

The likelihood-ratio (LR) chi-square test was chosen to assess the goodness-of-fit of the random effects using a *P*-value < 0.05 as significant. LR is calculated as the difference in the −2 log likelihood of the restricted model for each random effect relative to the full model. Under the null hypothesis, LR is expected to be distributed as $\chi_{q}^{2}$ with *q* degrees of freedom given by the difference in the numbers of parameters between the two models. A higher value of LR with respect to $\chi_{q}^{2}$ indicates that the estimated variance component is significant.

The fraction of the total variation due to additive and family variance components was estimated through the formulas for individual ($h_{i}^{2}$) and family mean ($h_{f}^{2}$) heritabilities, respectively. For the annual growth rhythm models and for the individual treatment tests, heritabilities were estimated as follows:

$${\hat{h}}_{i}^{2}=\frac{{\hat{V}}_{A}}{{\hat{V}}_{P}}=\frac{3{\hat{\sigma }}_{f}^{2}}{{\hat{\sigma }}_{f}^{2}+{\hat{\sigma }}_{e}^{2}}\;\mathrm{and}$$ (5)

$${\hat{h}}_{f}^{2}=\frac{{\hat{\sigma }}_{f}^{2}}{{\hat{\sigma }}_{f}^{2}+\frac{{\hat{\sigma }}_{e}^{2}}{b}},$$ (6)

where $V_{A}^{2}$ indicates the additive genetic variance, which was assumed to be ${3\sigma }_{f}^{2}$ to avoid overestimation of the additive variance [62], $V_{P}^{2}$ indicates the phenotypic variance, $\sigma_{f}^{2}$ indicates the family variance, $\sigma_{e}^{2}$ indicates the residual variance and *b* indicates the harmonic mean of the numbers of trees per family. For non-continuous variables, the residual variance of the GLIMMIX procedure was set to π^2^/3 [63]. The approximate standard errors for heritability estimates were calculated using the delta method [64].

Coefficients of additive genetic variance (*CV*_*A*_) were estimated to make possible the comparisons among continuous variables, for which the $V_{A}^{2}$ values were estimated as a function of the units used:

$${\hat{CV}}_{A}=100\frac{\sqrt{{\hat{\sigma }}_{A}^{2}}}{\mu },$$ (7)

where *μ* is the observed phenotypic mean of the trait. For those traits recorded as scores, the estimation of *CV*_*A*_ was not applicable due to the transformation of the outcome defined by the link function of the GLIMMIX procedure.

The individual tree phenotypic correlations (*r*_*p*_) between all pairs of traits were estimated for the two experiments. Moreover, in the drought-stress trial family mean correlations between treatments were also estimated. Pearson correlation coefficients were computed using the CORR procedure in SAS.

Additive genetic correlations (*r*_*A*_) were estimated between traits according the formula:

$$r_{A}=\frac{{{\hat{cov}}_{A}}_{\left(x,y\right)}}{{\hat{\sigma }}_{x}{\hat{\sigma }}_{y}},$$ (8)

where *σ*_*x*_ and *σ*_*y*_ indicate the square roots of the corresponding family variance components for traits *x* and *y*, respectively, and ${{cov}_{A}}_{\left(x,y\right)}$ is the family covariance component between the two traits estimated as:

$${\hat{COV}}_{A(x,y)}=\frac{{\hat{\sigma }}_{Z}^{2}-\left({\hat{\sigma }}_{x}^{2}+{\hat{\sigma }}_{y}^{2}\right)}{2},$$ (9)

where *z* is a synthetic variable created as the sum of variables *x* and *y*, and $\sigma_{z}^{2}$ is the family variance of that variable.

Approximate standard errors of *r*_*A*_ were computed using the formula suggested by Falconer [65]:

$$SE\left(r_{n}\right)=\frac{1-r_{gA}^{2}}{\sqrt{2}}\sqrt{\frac{SE\left(h_{x}^{2}\right)SE\left(h_{y}^{2}\right)}{h_{x}^{2}h_{y}^{2}}}$$ (10)

where $SE(h_{x}^{2})$ and $SE(h_{y}^{2})$ are the approximate standard errors of the individual heritabilities of the traits *x* and *y*, respectively.

For the statistical models in which population was considered as random effect, *Q*_*ST*_ was estimated according to Spitze [29]:

$${\hat{Q}}_{ST}=\frac{\sigma_{p}^{2}}{\sigma_{p}^{2}+2(3\sigma_{f}^{2})},$$ (11)

where $\sigma_{p}^{2}$ is the variance among populations. Approximate standard errors of *Q*_*ST*_ were estimated using the delta method [64].

Furthermore, relationships between measured traits and climatic parameters, shown in Table 1, were analyzed using a phenotypic correlation based on BLUPs estimated for each population.

### Molecular genetic analysis

All of the samples from the original mother trees selected from each stand included in this study were previously genotyped with nine microsatellite loci, and their genetic structure was analyzed by Fernández-Cruz and Fernández-López [38]. The ancestry values (q) at K = 2 (North Iberian and Mediterranean gene pools) were estimated with STRUCTURE 2.3.4 [66] using the global data of all of the individuals included in the mentioned study, which included 904 samples collected from 29 natural or naturalized stands.

Genotype data corresponding to the individuals of the populations included in each trial were reanalyzed to study the *F*_*ST*_ values [67] between all possible pairs among the nine stands. *F*_*ST*_ values were calculated with AMOVA [68, 69] using GENODIVE 2.0b25 [70], and the corresponding significance tests were based on 10,000 permutations. Other specific details of the methodology employed for the molecular analysis are based on Fernández-Cruz and Fernández-López [38].

### Comparisons between molecular and quantitative trait variation

The difference between *Q*_*ST*_ and *F*_*ST*_ was evaluated following the method proposed by Gilbert and Whitlock [71], which was a modification of the method described by Whitlock and Guillaume [72] for unbalanced half-sib designs. This method is based on a simulation resampling approach to statistically test the difference between *Q*_*ST*_ and *F*_*ST*_, assuming that the null hypothesis is *Q*_*ST*_ = *F*_*ST*_. Mean *F*_*ST*_ and *Q*_*ST*_ for each trait are resampled and the confidence intervals of the difference between both are estimated from the 2.5% and 97.5% quantiles of the bootstrap distribution. For each estimate, the method uses 10,000 bootstrap replicates. We used the R package QstFstComp in R 3.1.0 to implement the method [71].

To evaluate the relationship between the quantitative traits variation and the ancestry of the populations, Pearson correlations were determined between the ancestry values (q) in the Mediterranean cluster provided by Fernández-Cruz and Fernández-López [38] for each of the sampled trees of the nine stands and the BLUPs of their progenies estimated for each trait in the corresponding mixed model.

## Results

### Annual growth rhythm experiment

#### Genetic variation among populations

The results of the quantitative analysis for the studied traits in the 3 years of the annual growth rhythm experiment showed that population variance was significant for all traits, except for RCD, and that this significance was greater in the last year (Table 2). These increments of significance over time were more evident for S, BS and H, and for the latter, the effect of population was significant only in the last year. Family variance was significant for all traits except for STR, and contrary to that observed for population variance, its estimates were lower in the last year of evaluation, mainly for BS, H and S.

**Table 2:** Main results for the mixed-model analysis of the annual growth rhythm experiment. F tests for the fixed effect of block (*B*), estimates of variance components of the random effects and their significance based on likelihood ratio tests, individual (${\hat{h}}_{i}^{2}$) and family mean (${\hat{h}}_{f}^{2}$) heritability estimates and their standard errors, coefficients of additive genetic variance (${\hat{CV}}_{A}\%$), and values of phenotypic differentiation among populations (${\hat{Q}}_{ST}$) and their standard errors.

	*B*(*F*_19,146_)^a^	${\hat{\sigma }}_{p}^{2}$	${\hat{\sigma }}_{f}^{2}$	${\hat{\sigma }}_{e}^{2}$	${\hat{h}}_{i}^{2}$	${\hat{h}}_{f}^{2}$	${\hat{CV}}_{A}$	${\hat{Q}}_{ST}$
TF07	2.55**	0.51***	0.34***	3.29***	0.28 ± 0.08	0.61 ± 0.03	na	0.20 ±0.08
TF08	2.35**	0.94***	0.28***	3.29***	0.24 ± 0.06	0.57 ± 0.02	na	0.36 ±0.06
LF07	3.18***	0.61***	0.55***	3.29***	0.43 ± 0.11	0.72 ± 0.04	na	0.16 ±0.11
LF08	2.88***	1.16***	0.37***	3.29***	0.3 ± 0.07	0.63 ± 0.03	na	0.35 ±0.07
BS07	2.45^ns^	0.23**	0.35***	3.29***	0.29 ± 0.08	0.62 ± 0.03	na	0.1 ±0.09
BS08	0.18**	0.29***	0.17**	3.29***	0.16 ± 0.06	0.46 ± 0.03	na	0.22 ±0.09
H06	11.79 ***	0.59^ns^	52.11***	160.5***	0.74 ± 0.13	0.83 ± 0.04	61.9	—
H07	7.68***	7.04^ns^	53.05***	438.8***	0.32 ± 0.08	0.65 ± 0.03	29.4	—
H08	4.99***	45.36**	23.43*	817.7***	0.08 ± 0.05	0.31 ± 0.02	8.7	0.24 ±0.08
RCD07	2.84**	0^ns^	0.024***	0.139***	0.45 ± 0.09	0.73 ± 0.03	15.4	—
RCD08	38.9***	0^ns^	1.46***	19.64***	0.21 ± 0.08	0.53 ± 0.03	29.1	—
STR08	3.67***	0.062*	0.006^ns^	3.29***	—	—	—	—
AD08	2.36**	0.13*	0.11*	3.29***	0.1 ± 0.05	0.34 ± 0.02	na	0.16 ±0.1
S07	3.28***	0.267**	0.175***	3.29***	0.23 ± 0.04	0.59 ± 0.02	na	0.1 ±0.05
S08	4.87***	0.159***	0.145**	3.29***	0.12 ± 0.05	0.39 ± 0.03	na	0.16 ±0.09

Keys to variances: $\sigma_{p}^{2},$ population; $\sigma_{f}^{2},$ family; $\sigma_{e}^{2},$ error

*TF*, terminal flushing; *LF*, lateral flushing; *BS*, bud set; *H*, height; *RDC*, root collar diameter; *STR*, straightness; *AD*, apical dominance; *S*, survival; *06*, year 2006; *07*, year 2007; *08*, year 2008

Significance levels

*** *p* < 0.001

** *p* < 0.01

* *p* < 0.05

*ns*, not significant

na, not applicable

^a^F (df between groups, df within groups)

**Fig 2:**
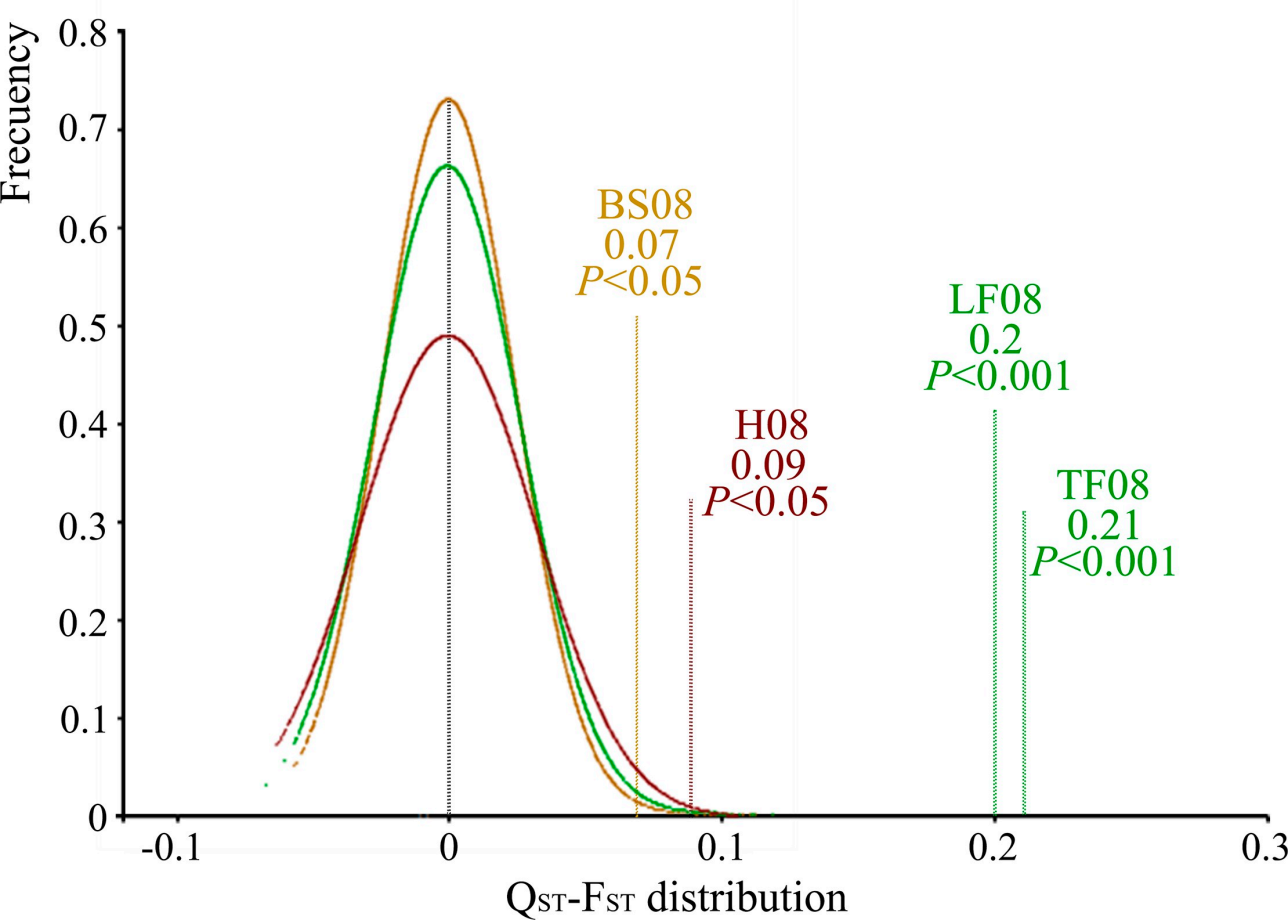
The simulated distributions (color curves) of *Q*_*ST*_*–F*_*ST*_ expected under neutrality and the observed estimates of *Q*_*ST*_*–F*_*ST*_ for the traits measured in 2008 which showed significant differences between both parameters in the annual growth rhythm experiment. The distributions of *Q*_*ST*_−*F*_*ST*_ was simulated following the method of Gilbert and Whitlock [71] which provides 10,000 simulated estimates of this difference for each trait. The *P*-value was obtained by comparison of the observed *Q*_*ST*_−*F*_*ST*_ to the quantile of the corresponding simulated distribution. *TF*, terminal flushing; *LF*, lateral flushing; *BS*, bud set; *H*, height. Note: The simulated distribution of TF and LF is overlapping.

The TF and LF measured in 2008 showed the greatest *Q*_*ST*_ (0.35–0.36) followed by H in 2008 (0.24), BS in 2008 (0.22), TF in 2007 (0.20), S in 2008 (0.19), AD in 2008 (0.16) and LF in 2007 (0.16) (Table 2). The rest of the variables with a significant population variance had a lower *Q*_*ST*_ (< 0.16). The overall *F*_*ST*_ was 0.15 (p < 0.001). For the pairwise *F*_*ST*_ values (S1 Table) the greatest *F*_*ST*_ was between CR3 and CR6 (0.263). Moreover, these both populations showed a high differentiation compared with several populations: CR3 with CR4 (0.227), CR9 (0.205) and CR14 (0.202), and CR6 with CR1 (0.209), CR2 (0.219), CR4 (0.21) and CR14 (0.22). However, a very low differentiation was detected between CR12 and CR4 (0.029) and between CR1 and CR2 (0.049), as we expected because of the geographical proximity of these two pairs of populations (see Fig 1). The difference between *Q*_*ST*_ and *F*_*ST*_ was significant for the evaluations made in 2008 for flushing, BS and H, and in all cases, the phenotypic differentiation among these traits was greater than expected under neutrality (Fig 2).

**Fig 3:**
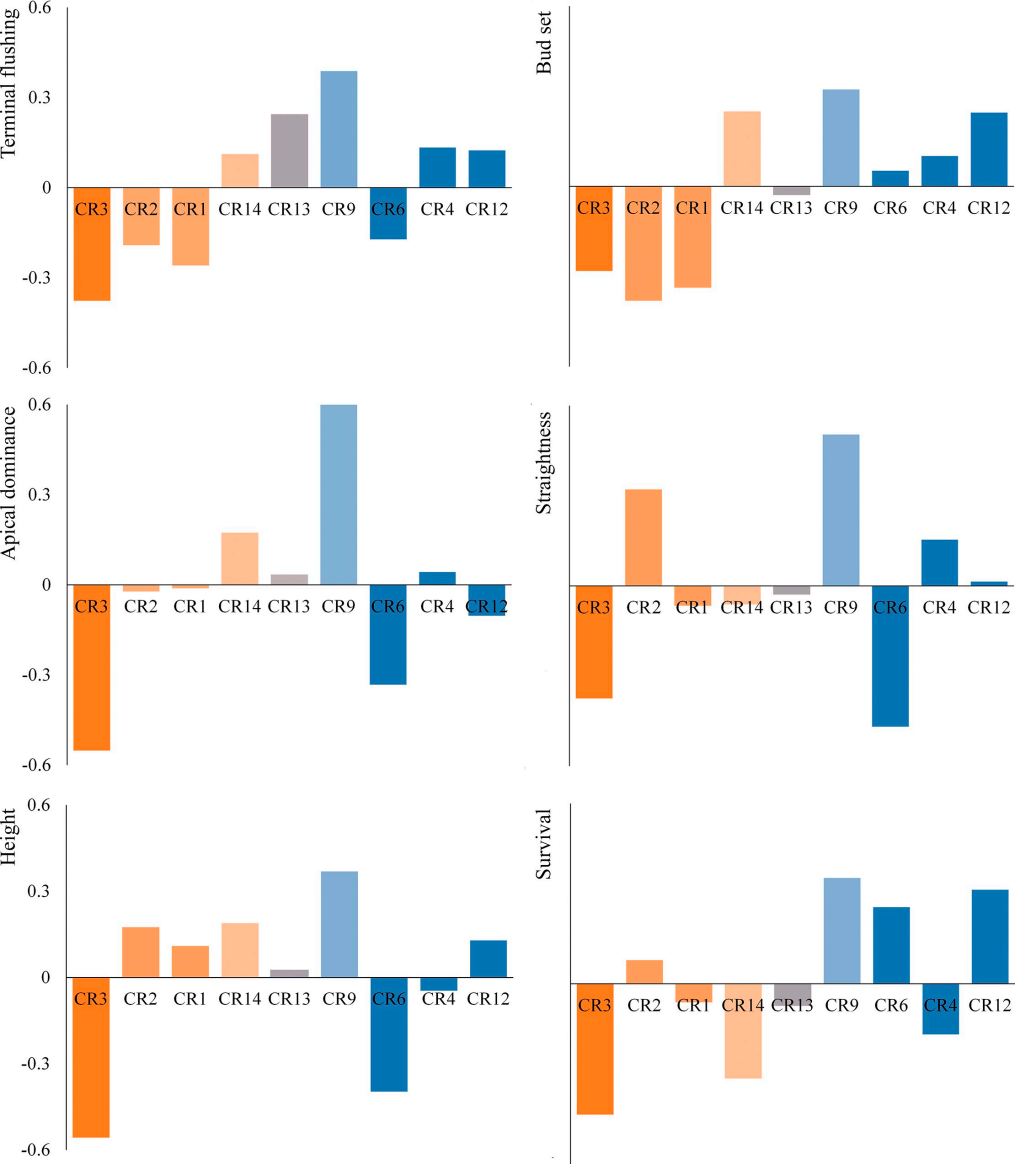
Standardized best linear unbiased predictors of the populations for the traits measured in 2008 that showed a significant population variance in the annual growth rhythm experiment. For each trait, positive values were standardized with respect to the sum of the positive values, and the same process was repeated with the negative values. Populations were sorted according to their membership coefficients for the Mediterranean cluster. Blue and orange color gradients represent the membership proportions of Northern Iberian Peninsula gene pool and Mediterranean gene pool, respectively. The most intense colors represent the highest ancestry values for each cluster. CR13 is represented by a mixture of the lightest colors representing both gene pools.

The BLUP values of the populations for the traits evaluated in 2008 that showed a significant population variance evidenced a practically opposite behavior between the populations of CR9 and CR3 (Fig 3). CR9 showed the latest flushing and BS, and the greatest values for H, tree form traits and S. CR3 had the lowest values for H, AD and S, and it was one of the populations with earliest flushing and BS, along with CR1 and CR2.

The geographic variation of quantitative traits among populations was confirmed by the significant correlations of the xerothermic index and the latitude of the populations’ origin with flushing traits [(−0.67)–(−0.76) and (0.84)–(0.88), respectively], BS (−0.76 and 0.89, respectively), H (−0.77 and 0.69, respectively) and AD (−0.74 and 0.74, respectively), all measured in 2008 (Table 3). These relationships indicated that northern populations with low xerothermic indices showed a late flushing, late BS, faster growth and a tendency to a better conformation than the central and southern populations. Moreover, the highly significant negative correlation found between the ancestry in the Mediterranean gene pool and the traits of TF and LF [(−0.37)–(−0.34)] and BS (−0.28) indicate that phenology are clearly related to the genetic structure of the Iberian populations that had been identified using microsatellites (S2 Fig).

**Table 3:** Pearson correlations between the values of some climatic and geographical parameters and the population means (N = 9), and between the Mediterranean ancestry values for selected trees of each stand and the progeny means (N = 192), for traits that showed a significant population variance in the annual growth rhythm experiment.

	TF07	TF08	LF07	LF08	BS08	H08	AD08	S08
ATD (°C)	*ns*	*ns*	*ns*	*ns*	*ns*	0.66*	*ns*	*ns*
*X*_*i*_	-0.67*	-0.76*	-0.69*	-0.70*	-0.76*	-0.77*	-0.74*	*ns*
Latitude	0.85**	0.88**	0.84**	0.87**	0.89**	0.69*	0.74*	*ns*
K2Med	-0.34***	-0.34***	-0.37***	-0.32***	-0.28***	*ns*	*ns*	-0.35***

Origin data: ATD, difference between the absolute maximum and minimum temperatures; Xi, xerothermic index, K2MED, ancestry in the Mediterranean cluster at K = 2

*TF*, terminal flushing; *LF*, lateral flushing; *BS*, bud set; *H*, height; *AD*, apical dominance; *S*, survival; *08*, year 2008

Significance levels:

*** *p* < 0.001

** *p* < 0.01

* *p* < 0.05

*ns*, not significant

#### Genetic variation within populations

Estimated $h_{i}^{2}$ values were moderate for flushing (0.24–0.43), ranged from low to moderate for BS (0.16–0.29) and S (0.12–0.25), from low to high for growth (0.08–0.74), and were very low for AD (0.1) (Table 2). In all cases the lowest estimates were obtained in 2008, and the most marked difference between years was for H. The $h_{f}^{2}$ values were greater than $h_{i}^{2}$ in all of the cases, ranging from 0.31 for AD in 2008 to 0.83 for H in 2006, and they followed the same trend as $h_{i}^{2}$ estimates. The *CV*_*A*_ values, estimated only for the continuous variables, ranged from 8.7 to 61.9% for H and from 15.4 to 29.1% for RCD.

The values of additive variance and their coefficients obtained for each provenance for the main traits measured in 2008 showed that CR2 and CR3 were the most variable stands for TF and LF (S2 Table). CR2 also showed the highest additive variance and *CV*_*A*_ values for H and RDC. CR9 and CR13 had the lowest values of additive variance for TF and LF, respectively, and CR1 for BS and growth traits. Moreover, the $h_{i}^{2}$ values were highly variable among stands for phenology and growth, with estimates that ranged from very low to high. In any case, all these results should be considered with caution because of the unbalanced data obtained for some stands.

#### Correlations between traits

Very high and high phenotypic correlations were observed between TF and LF (0.72–0.89) and H and RCD (0.55–0.69) measured in the same year (S3 Table). For the same type traits measured in different years, phenotypic correlations were strong between flushing traits (0.42–0.45) and were even stronger between growth traits (0.44–0.77), except for H in 2006, which showed only a moderate to high correlation with the growth traits measured in 2008 (0.39–0.45). In both 2007 and 2008, flushing had significant positive and moderate phenotypic correlations with growth, which were slightly higher for H (0.27–0.48) than for RCD (0.16–0.33). Positive and weak correlations were found between BS and flushing (0.11–0.2) for the same year, and between BS and growth, in this case they were slightly greater for RCD (0.13–0.28) than for H (0.09–0.23). Moreover, the AD measured in 2008 showed a significant negative and a weak positive correlation with flushing in 2008 [(−0.15)–(−0.16)] and BS [(−0.18)–(−0.21)], as well as a negative moderate correlation with H measured in 2008 (−0.31).

Estimated *r*_*A*_ values were greater than those estimated for phenotypic correlations in all cases (S3 Table). The lowest estimates for this parameter were dismissed because of their high standard error. These results confirmed the positive correlations observed between flushing and growth (0.34–0.66) and between flushing and BS (0.31–0.46), and the negative correlation between AD and H measured in 2008 (-0.4). This indicated that trees with late flushing had greater H values and a later BS, and that the tallest trees were more likely to have high AD values.

### Periodic drought-stress experiment

#### Genetic variation among populations

**Fig 4:**
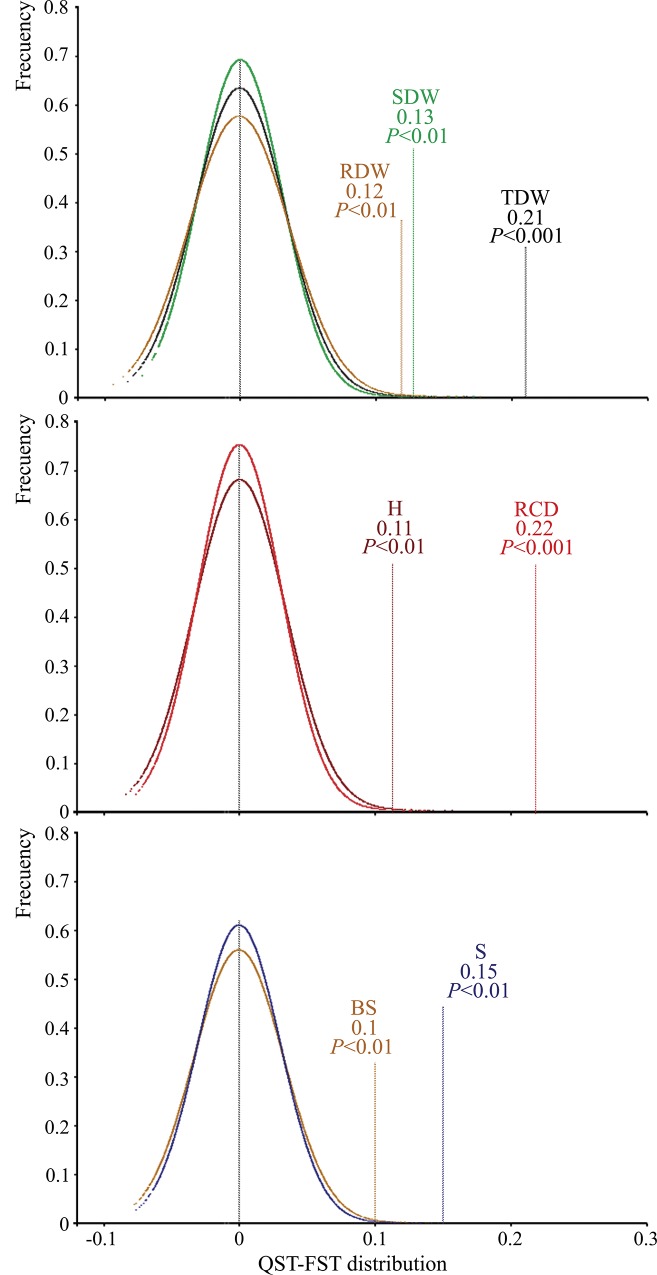
The simulated distributions (color curves) of *Q*_*ST*_*–F*_*ST*_ expected under neutrality and the observed estimates of *Q*_*ST*_*–F*_*ST*_ for the traits measured in T27D which showed significant differences between both parameters in the drought-stress experiment. The distributions of *Q*_*ST*_*–F*_*ST*_ was simulated following the method of Gilbert and Whitlock [71] which provides 10,000 simulated estimates of this difference for each trait. The *P*-value was obtained by comparison of the observed *Q*_*ST*_*–F*_*ST*_ to the quantile of the corresponding simulated distribution. *SDW*, stem dry weight; *RDW*, root dry weight; *LDW*, leaf dry weight; *TDW*, total dry weight; *RDC*, root collar diameter; *H*, height; *BS*, bud set; *S*, survival.

Different results were obtained from the analysis model for each treatment separately and for the corresponding genetic parameter estimates (Table 4). The random effect of population was significant for SDW, RDW and TDW, RCD, H, BS and S, measured at the end of the experiment in both treatments. Moreover, the among-population variance was also significant for LDW, DEF and DA in T27D. The estimated values of *Q*_*ST*_ indicated that there was a higher population differentiation in T27D than in T21W for SDW (0.28), RDW (0.27), TDW (0.36), RCD (0.37) and S (0.30), and similar values in both treatments for H (0.26–0.27) and BS (0.25–0.28). In all T27D cases, the *Q*_*ST*_ estimates were significantly higher than the *F*_*ST*_ value (Fig 4).

**Table 4:** Main results for the mixed analysis for each treatment of the periodic drought-stress experiment. F tests for the fixed effect of block (*B*), and for the covariates of days for germination (*DG*) and height at the beginning of the experiment (*H2*), estimates of variance components of the random effects and their significance based on likelihood ratio tests, individual (${\hat{h}}_{i}^{2}$) and family means (${\hat{h}}_{f}^{2}$) heritability estimates and their standard errors, coefficients of additive genetic variance (${\hat{CV}}_{A}\%$), and values of phenotypic differentiation among populations a (${\hat{Q}}_{ST}$) and their standard errors.

		*B* (*F*_19,152_)^a^	*DG* (*F*_1,896_)	*H2* (*F*_1,896_)	${\hat{\sigma }}_{p}^{2}$	${\hat{\sigma }}_{f}^{2}$	${\hat{\sigma }}_{e}^{2}$	${\hat{h}}_{i}^{2}$	${\hat{h}}_{f}^{2}$	${\hat{CV}}_{A}$	${\hat{Q}}_{ST}$
T21W	SDW	3.84***	2.78^ns^	2043.5***	1.71***	1.09**	24.04***	0.13±0.05	0.5±0.02	30.05	0.21±0.09
	RDW	3.42***	1.15^ns^	883.9***	1.09***	0.873***	17.22***	0.15±0.02	0.48±0.02	24.21	0.17±0.11
	LDW	3.22***	7.31**	754.1***	0.125^ns^	0.204*	7.82***	0.08±0.04	0.32±0.06	18.77	—
	TDW	4.72***	1.23^ns^	1529***	7.43***	5.7**	128.9***	0.12±0.04	0.41±0.04	25.14	0.18±0.1
	RCD	0.85^ns^	4.5*	298.9***	0.0005*	0^ns^	0.033***	—	—	—	—
	DEF	2.48***	3.1^ns^	3.6 ^ns^	0 ^ns^	0^ns^	3.29***	—	—	—	—
	NSB	0.47^ns^	1.27^ns^	0.17^ns^	0^ns^	0 ^ns^	0.284***	—	—	—	—
	H	1.97*	1.58^ns^	3547.4***	46.46***	21.02**	442.8***	0.14±0.05	0.46±0.02	17.45	0.27±0.08
	BS	6.95***	1.17^ns^	381.75***	0.257***	0.108**	3.29***	0.1±0.04	0.37±0.03	na	0.28±0.07
	DA	4.35***	0.97^ns^	115.3***	0.046^ns^	0.08^ns^	3.29***	—	—	—	
	S	0.37^ns^	2.26^ns^	12.41***	0.872**	0.373 ^ns^	3.29***	—	—	—	—
T27D	SDW	2.77***	3.47^ns^	1459.8***	1.174***	0.494***	6.628***	0.21±0.06	0.57±0.02	29.36	0.28±0.07
	RDW	5.9***	1.96^ns^	251.4***	0.831***	0.363***	3.524***	0.28±0.07	0.65±0.02	25.42	0.27±0.07
	LDW	7.37***	0.46^ns^	83.3***	0.098**	0^ns^	2.901***	—	—	—	—
	TDW	8.54***	2.87^ns^	617.1***	5.59**	1.6**	28.22***	0.15±0.03	0.43±0.04	20.93	0.36±0.05
	RCD	1.44^ns^	0.35^ns^	285.58***	0.005**	0.001*	0.034***	0.11±0.05	0.41±0.02	9.39	0.37±0.05
	DEF	19.4***	0.52^ns^	46.48***	0.101*	0.05^ns^	3.29***	—	—	—	—
	NSB	1.71*	0.25^ns^	2.68^ns^	0.0006^ns^	0.028*	0.372***	0.21±0.11	0.58±0.04	16.12	—
	H	1.05^ns^	0.16^ns^	25057.4***	62.04***	29.03**	356.5***	0.23±0.07	0.6±0.02	20.58	0.26±0.08
	BS	1.81*	0.06^ns^	335.84***	0.264***	0.133***	3.29***	0.12±0.05	0.35±0.02	na	0.25±0.07
	DA	1.62*	0.07^ns^	78.1***	0.073*	0.054^ns^	3.29**	—	—	—	—
	S	3.61***	3.66^ns^	0.9^ns^	0.645***	0.255*	3.29***	0.22±0.06	0.5±0.02	na	0.30±0.06

Keys to variances: $\sigma_{p}^{2}$ population, $\sigma_{f}^{2}$ family, $\sigma_{e}^{2}$ error

*T21W*, well-watered treatment; *T27D*, drought-stress treatment; *SDW*, stem dry weight; *RDW*, root dry weight; *LWD*, leaf dry weight; *TWD*, total dry weight; *RCD*, root collar diameter; *DEF*, defoliation; *NSB* number of secondary branches; *H*, height; *BS*, bud set; *DA*, dry apex; *S*, survival

na, not applicable

Significance levels:

*** *p* < 0.001

** *p* < 0.01

* *p* < 0.05

*ns*, not significant

^a^: df between groups, df within groups

A significant positive relationship between TDW and the ancestry in the Mediterranean gene pool was observed for both treatments, with a greater significance in T27D (0.39, *P* < 0.001; data not shown) than in T21W (0.34, *P* < 0.01; data not shown). For this correlation, the root system contributed more than the stem and showed a larger value in T27D (0.42, *P* < 0.001) than in T21W (0.36, *P* < 0.01), indicating a greater development of the root system in the central and southern populations under water stress (S2 Fig). No correlation was found between S and the Mediterranean cluster in T21W but a slightly significant correlation was observed in T27D (0.27, *P* < 0.05; data not shown). No significant correlations were found for the traits evaluated against climatic and geographical parameters.

In the combined analysis, the fixed effect of treatment was highly significant for all of the traits but the fixed population × treatment interaction was significant only for SDW, RDW, TDW, RCD, DEF, BS and S (Table 5). The fixed effect of the population was significant for all of the traits, except NSB and DA, and the family variation showed significant values for SDW, RDW, TDW, H, BS and S.

**Table 5:** Main results for the mixed analysis combined for both well-watered and periodic drought-stress treatments. F tests for the fixed effect of treatment (*T*), block within treatment [*B*(*T*)], population (*P*), population × treatment interaction (P*T), for the covariates of days for germination (*DG*) and height at the beginning of the experiment (*H2*), estimates of variance components of the random effects and their significance based on likelihood ratio tests, individual (${\hat{h}}_{i}^{2}$) and family means (${\hat{h}}_{f}^{2}$) heritability estimates, and their standard errors and coefficients of additive genetic variance (${\hat{CV}}_{A}\%$).

	*T* (*F*_1,80_)^a^	*B(T)* (*F*_38,1709_)	*P* (*F*_8,80_)	P*T (*F*_8,80_)	*DG* (*F*_1,1709_)	*H2* (*F*_1,1709_)	${\hat{\sigma }}_{f}^{2}$	${\hat{\sigma }}_{ft}^{2}$	${\hat{\sigma }}_{e}^{2}$	${\hat{CV}}_{A}$
SDW	234.58	3.1***	2.47*	3.55**	1.13^ns^	234.58***	0.082*	0.142*	7.589***	9.69
RDW	258.18***	2.1***	4.33***	2.26*	0.81^ns^	764.7***	0.337***	0.433***	7.743***	19.15
LDW	279.81***	2.46***	3.08**	1.46^ns^	3.22^ns^	598.17***	0.048^ns^	0.066^ns^	4.231***	—
TDW	324.65***	2.91***	3.74***	2.71*	0.85^ns^	1394***	1.02**	1.66**	47.6***	12.68
RCD	111.63***	0.98^ns^	2.84**	3.67**	3.84^ns^	482.78***	0.001^ns^	0.002^ns^	0.027***	—
DEF	1978.2***	4.31***	9.08**	3.01**	0.12^ns^	21.2***	0.01^ns^	0^ns^	3.29***	—
NSB	73.46***	1.93***	1.62^ns^	0.87^ns^	0.17^ns^	1.58^ns^	0.006^ns^	0.015**	0.461***	—
H	57.27***	4.38***	10.35***	1.16^ns^	0.7^ns^	9833.05***	26.82***	21.24***	410.9***	19.6
BS	10.28**	4.31***	9.1***	2.45*	0.6^ns^	767.8***	00.154***	0.122***	3.29***	na
DA	65.14***	2.43***	3.04***	0.67^ns^	0.12^ns^	119.05***	0.072^ns^	0.014^ns^	3.29***	—
S	60.48***	356***	3.86***	3.44**	6.25*	4.43*	0.26***	0.18**	3.29***	na

Keys to variances: $\sigma_{f}^{2},$ family; $\sigma_{ft}^{2},$ family × treatment interaction; $\sigma_{e}^{2},$ error

*SDW*, stem dry weight; *RDW*, root dry weight; *LDW*, leaf dry weight; *TWD*, total dry weight; *RCD*, root collar diameter; *DEF*, defoliation; *NSB* number of secondary branches; *H*, height; *BS*, bud set; *DA*, dry apex; *S*, survival

na, not applicable

Significance levels:

*** *p* < 0.001

** *p* < 0.01

* *p* < 0.05

*ns*, not significant

^a^: df between groups, df within groups

**Fig 5:**
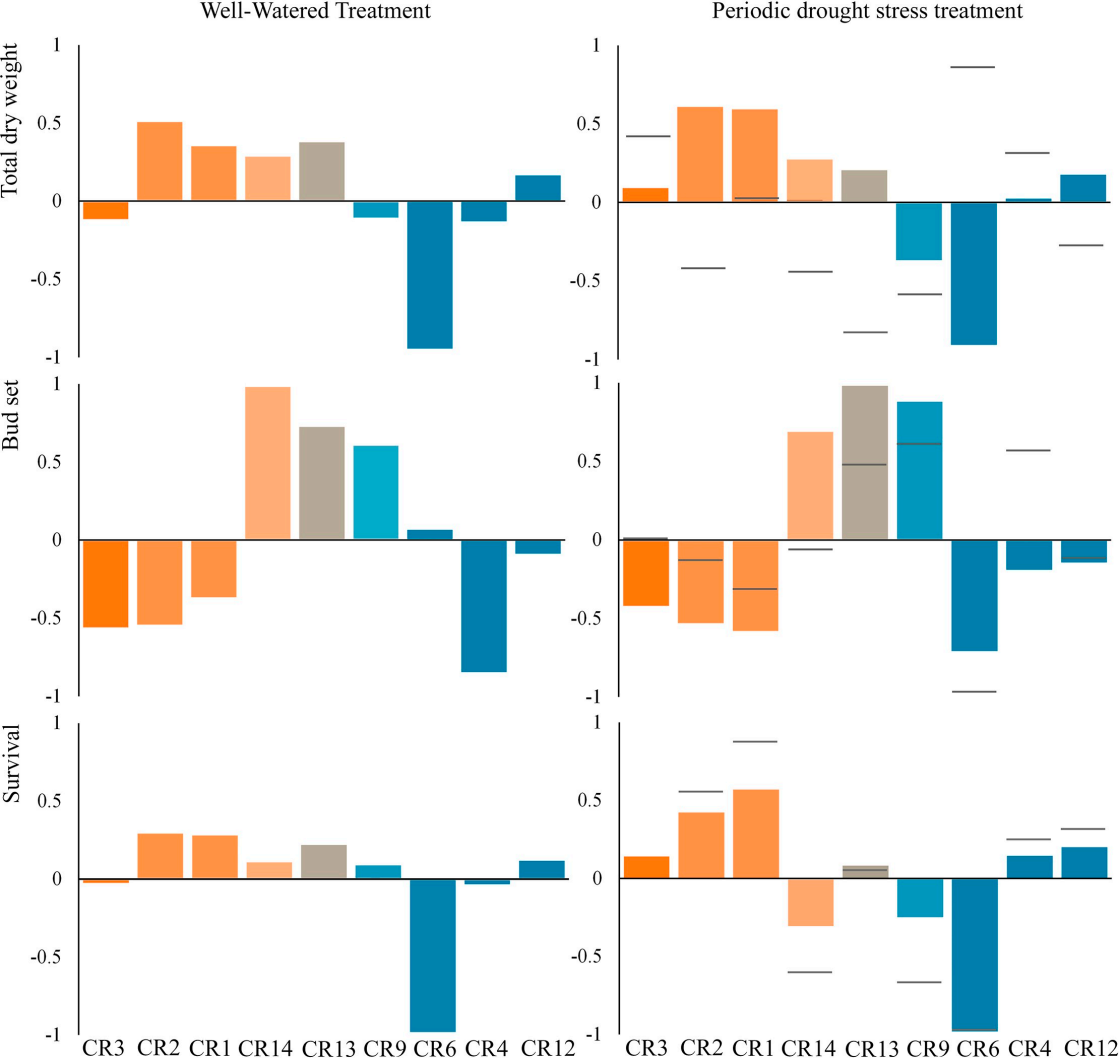
Standardized best linear unbiased predictors of *C*. *sativa* populations (color bars) for each treatment separately, and the standardized differences in the population means between treatments (horizontal bars), for total dry weight, bud set and survival. Before standardizing the differences between means, the deviation of each population with respect to the average of these differences were estimated. Blue and orange color gradients represent the membership proportions (darkest colors = q > 0.9) of the populations belonging to the Northern Iberian Peninsula and Mediterranean gene pools, respectively. CR13 is represented by a mixture of the lightest colors representing both gene pools. *TDW* = total dry weight; *BS* = bud set; *S* = survival; *T1* = control treatment; *T2* = drought-stress treatment.

There were notable differences between treatments for the standardized BLUPs of populations (Fig 5) and families within populations (S3 Fig) in those traits affected by the interactions of treatment × population and treatment x family. For TDW, the ranking of populations was similar in both trials, with the CR6 population having the lowest values. The Central West Iberian populations, CR1 and CR2, and the northern populations of CR12, CR13 and CR14 showed positive BLUP values in both treatments. The populations with less TDW loss and, consequently, with positive values for the standardized differences in the means between treatments were CR6, the southern CR3 population and CR4 (Fig 5). As we expected, because of annual growth experiment results, the central (CR1 and CR2) and southern (CR3) populations underwent early BS, and this observation was the same in both treatments. However, there was an important change in the ranking of populations in T27D. Although the general observation for this trait is that trees had an earlier BS under drought-stress (data not shown), CR4, CR9 and CR13 showed a later BS under drought-stress, while CR6 showed the earliest BS. Finally, central and southern populations had the best S values in T27D, while CR6, CR9 and CR14 had the worst.

#### Genetic variation within populations

The family variance was significant for SDW, RDW, TDW, H, BS and S in both treatments, and it had low significances for LDW in T21W and for NSB and RCD in T27D (Table 4). The $h_{i}^{2}$ estimated for dry weight traits, H and BS were greater in T27D (0.15–0.28, 0.23 and 0.12, respectively) than in T21W (0.08–0.15, 0.14 and 0.1, respectively) and $h_{f}^{2}$ followed the same pattern but with higher values. The highest *CV*_*A*_ values were obtained for SDW (29.4–30%), RDW (24.2–25.4%), TDW, which showed higher values in T21W (25.1%) than in T27D (20.3%), and H, which were lower in T21W (17.4%) than in T27D (20.6%).

In the combined analysis, the same traits for which the family variance was significant also had significant family × treatment interactions along with NSB (Table 5). An important change in the ranking of families in T27D can be observed for TDW, BS and S (Fig 5).

#### Correlations between traits and between treatments

Positive correlations for BS with H (0.58), and with SDW and LDW (0.29–0.38) indicated that plants with later BS underwent greater growth and, consequently, had higher dry weights for the aerial parts of the plants. Plants with earlier BS also showed a greater number of DAs (correlation of −0.51). DEF showed a positive correlation with axillary buds sprouting (0.49) and an important negative correlation with LDW (-0.39). Moreover, S had a moderate positive correlation with SDW and RDW. Finally, as expected, a very high positive phenotypic correlation was observed between SDW and H (0.76), and high individual-tree phenotypic correlations were found between dry weight and growth (0.39–0.54) in the combined analysis of treatments (S4 Table).

No correlations of family means between treatments were found for DEF, indicating that the behavior of the families for this trait was completely different under drought conditions compared with that observed in the control treatment, even though the differences were not reflected in the statistical analysis. Additionally, for families between treatments the LDW, RCD and NSB values showed moderately positive correlations (0.25–0.27). The highest correlations were for H (0.73) and for SDW and RDW (057–0.65)

## Discussion

This study provides a broad view of the genetic variation among and within nine Iberian populations for several important adaptive traits. It reports the first results of a drought-stress experiment in this area, and it is the first study to compare quantitative and molecular data to provide greater information on the role of natural selection on the geographic structure of the studied populations and to relate this structure with the gene pools identified in the Iberian Peninsula.

### The genetic variation observed among populations reveals a geographic structure associated with differential effects of natural selection

The results obtained from our study of genetic variation confirmed the high level of genetic differentiation among Iberian chestnut populations for most adaptive traits related to annual growth rhythm and an increasing population effect for phenology and growth over the years, as previously reported by Míguez-Soto and Fernández-López [52] in six European and six geographically distant Spanish populations. This population differentiation followed a north–south, or wet–xeric, pattern as indicated the significant correlations of flushing, BS and H with the xerothermic index and the latitude of the origin of provenances. Clinal geographic pattern was reported previously for provenance trials of *C*. *sativa* in Spain, in which populations with low xerothermic indices and higher latitudes showed later flushing [49, 51, 52], later BS and faster growth [52]. This pattern was also observed in provenance trials of European populations from Spain, Italy and Greece for phenology and growth [50, 52] and for functional expressed sequence tag-SSR markers [73]. All these results suggest that natural selection had a major role in this population differentiation. In our study, the role of this evolutionary force was evaluated through *Q*_*ST*_*–F*_*ST*_ comparisons. In spite of potential biasing effects, such as phenotypic plasticity, environmental maternal effects, non-additive genetic interactions and other imprecisions in the *Q*_*ST*_ estimation that could affect the *Q*_*ST*_*–F*_*ST*_ comparisons [74], the significance that we reported using the Whitlock and Guillaume [72] method and the greater values for the *Q*_*ST*_*–F*_*ST*_ differences obtained in our study for flushing (0.20–0.21), compared with the average differences reported in most studies (0.12) [75], confirmed that neutral processes are insufficient to explain the observed phenotypic trait divergence and that a natural differential selection process had taken place. Our results confirm the expectation that drought is a powerful selective force explaining the limitations of the distribution range of this species in southern latitudes [49]. Southern populations flush early because their period of available water is confined mainly to spring and also undergo early growth cessation to reduce transpiration during the dry summer. However, the highest risk for northern populations growing in a humid climate is related to spring and autumn frosts. Therefore, a later initiation of growth avoids major frost damage, and in this sense, cold also acts as a selective force [51]. Similar results were found in other Fagaceae species [16, 17]

From the comparisons between T21W and T27D in the periodic drought-stress experiment, we observed that in the drought treatment analysis there was a more evident quantitative differentiation among populations than in the control treatment for SDW, RDW, TDW, RCD and S. H and BS did not show this clear difference between treatments in our study, probably because, during the experimental period, measurements were made when elongation was nearly completed and BS had started for some plants. Even so, in the combined analyses, the fixed factor of population × treatment was significant for BS, and an earlier growth cessation was observed in most provenances under drought conditions, which was similar to the results of Jensen and Hansen [76] for this trait in their greenhouse experiment with *Quercus robur*. Our observations indicated that, apart from photoperiod and temperature factors to which temperate trees are evolutionary adapted [77], drought seems to be the main cause of growth cessation during the summer season in *C*. *sativa*. In our study, no clinal variation was observed for the evaluated traits related to drought. However, the significant *Q*_*ST*_*–F*_*ST*_ differences estimated mainly in the T27D analysis of SDW, RDW, TDW, RCD and S indicate the existence of local adaptations through natural selection and different responses of populations to drought-stress for these traits.

### Iberian populations are grouped into two distinct ecotypes, mesophytic and xeric, showing different adaptive responses to water stress

Our annual growth study reported significant correlations between BLUP values of the progenies within each population for phenology and survival with the ancestry values in the Mediterranean gene pool of the original trees of each stand, which related the genetic structure of the studied populations through neutral markers to the phenotypic observations. Moreover, in the periodic drought-stress experiment, positive correlations were estimated between the ancestry values in the Mediterranean cluster with RDW, showing higher values and significance in the drought-stress treatment than in the control treatment, and also with S, obtaining significance only under the drought-stress treatment. These observations lead us to indicate the presence of different ecotypes [6] that evolved from conserved populations adapted to different ecological conditions. Two ecotypes, corresponding to the two gene pools identified in the Iberian Peninsula, are defined: Mediterranean or xeric ecotype and North or mesophytic ecotype.

Populations belonging to mesophytic ecotype, contrary to the xeric populations, showed a later phenology and higher survival in the annual growth rhythm experiment, although the BLUP values of some populations did not exactly follow this classification. The northern populations of CR6 and CR4, belonging to the North Iberian Peninsula gene pool, and CR14, belonging to the Mediterranean gene pool (Fig 1), showed behaviors opposite of those expected. The possible explanation for CR6, which had low values of BLUPs for flushing, and CR4, which had low S values, may be that both are marginal populations of recent recolonization, probably affected by genetic drift [38], and also both are coastal populations under low selective pressure due to climatic conditions. Moreover, the importance of the Mediterranean gene pool in CR14 could be explained by the introgression of introduced germplasm.

The result obtained in the drought-stress experiment indicated that, in general terms, xeric populations developed larger root systems under drought conditions, which is a characteristic adaptation to drought by forest trees [78]. The higher survival observed in xeric populations under water stress conditions in our study also supported previous suggestions relating to the greater adaptive ability of the central and southern Iberian populations to drought [49, 52]. The between treatment comparisons of the BLUP values of our study populations for the traits more affected by the drought-stress treatment provided additional information on the behavior of each population (Fig 5). The three Mediterranean populations from the central and southern Iberian Peninsula, CR3, CR1 and CR2 (Fig 1), showed early BS, and for the last two, this trait occurred comparatively earlier under drought-stress conditions. When the BLUPs of CR3, which had the greatest ancestry value in the Mediterranean cluster, were compared between treatments, this population showed a drought-adapted behavior, resulting in the development of a larger root system and only a low mortality rate. Moreover, CR1 and CR2 showed the highest S responses under drought conditions, and these results supported the idea that provenances from southern latitudes were more adapted to increasing drought conditions. The northern population of CR9 showed the opposite behavior compared with CR3, resulting in an important loss of TDW, later BS and the second highest mortality rate. However, we did not observe a common behavior for the Northern populations in the change of ranking between treatments, and, as we previously mentioned, it could be caused by the confounding factors associated with CR6, CR4 and CR14.

Despite the demonstrated introgression of germplasm in the Iberian Peninsula due to the introduction and movement of grafted nut varieties from other Mediterranean areas [38], our hypothesis of the existence of two ecotypes evolved from conserved populations is also supported by the evidence of several Pleistocene refugia of the *C*. *sativa* in the Iberian Peninsula [79, 80] and the reduced genetic flow between populations of this species [38, 40].

### The intra-population variability observed in Iberian chestnut populations indicates their evolutionary potential to environmental changes

In spite of the expected reduction in genetic variation within populations because of the effects of directional selection, which resulted from the extreme environments reported in our annual growth study and the limited gene flow of *C*. *sativa* [38], we found significant within population variation for most measured traits. Our study confirms that, as also was observed by Míguez-Soto and Fernández-López [52], flushing is under strong genetic control in *C*. *sativa* and is more heritable than other growth and stem-form traits. Moreover, the high *CV*_*A*_ values obtained in this research for growth and some drought-related traits, indicated their variability in relation to selection responses [81]. The significant effect of family × treatment estimated for SDW, RDW, TDW, NSB, H, BS and S in our drought-stress experiment indicated the existence of differences in the adaptability of families under drought conditions, as reflected in S3 Fig which showed the changes in the family rankings for TDW, BS and S. With respect to the estimated *CV*_*A*_ within populations, the unbalanced data for some provenances and our inability to estimate accurately this coefficient for most traits, hinder the ability to draw any conclusions and more specific studies are needed on this subject.

The individual phenotypic and additive genetic correlations between the measured traits in the annual growth experiment agreed, in general, with previous observations reported by Míguez-Soto and Fernández-López [52] for *C*. *sativa* in their Spanish and European progeny–provenance trials. Thus, the tallest trees were those that showed late flushing and late BS, and which had the best stem conformation. Moreover, individual tree phenotypic correlations carried out in the drought-stress experiment indicated that TDW is an adequate trait to infer common results with the other dry weight traits and H. Other observed correlations indicated that plants with a late BS undergo more growth and, consequently, have higher aerial part dry weights. Additionally, plants with an earlier BS showed greater numbers of DAs, indicating again the direct relationship between summer droughts and growth cessation. Finally, the moderate positive correlation between RDW and S suggested that the development of larger root systems is a strategy for the survival of the plants during drought conditions.

## Conclusions

In light of the results obtained in the present study, we can conclude that the high genetic differentiation detected among wild Iberian *C*. *sativa* populations responds both to a latitudinal clinal pattern, with drought acting as a main force in central and southern Mediterranean populations, and to an ecotypic variation due to the restricted or absent gene flow among these geographically distant populations. Populations of the Northern Iberian Peninsula gene pool, corresponding to the mesophytic ecotype, showed late flushing and bud set, and had a tendency to a higher growth. On the contrary, populations of the Mediterranean gene pool, corresponding to the xeric ecotype, showed an opposite pattern for these adaptive traits and a greater adaptability for certain drought-related traits. Finally, the high within population genetic variability provided in this study, mainly at loci related to phenology and growth, and the estimates obtained for the related genetic parameters for most measured traits, indicated the potential of this species to adapt to future climate changes.

The genetic information provided in this study, along with the previous genetic information available for this species, constituted an important resource for facilitating the delineation of breeding zones, with a general recommendation of avoiding the north–south movement of seeds, and additional information for the implementation of long-term conservation strategies. Moreover, the velocity of climate change, the increased disease mortality and the emergence of new pests, require rapid adaptive actions in the forest management of this species, and the present study reports valuable information for future related studies.

## Supporting information

S1 Fig **Evolution of the mean water weight (A) and the daily average temperatures (B) for the well-watered (solid line) and the periodic drought-stress (broken line) treatments.** Temperature was recorded by eight temperature and humidity sensors that were located at different locations in the two growth chambers.
(TIF)

S2 Fig **Correlation between the ancestry in the Mediterranean gene pool of the individuals from each population with the means of their corresponding progenies for the number of days to terminal flushing (A1) and to bud set (A2) in 2008 (*N* = 192) evaluated in the annual growth rhythm experiment, and with the progeny means of root dry weight measured in the well-watered treatment (B1) and in the periodic drought-stress treatment (B2) (*N* = 90).** All of the correlation coefficients were significant at *P* < 0.001, except for that of B1 (*P* < 0.01).
(TIF)

S3 Fig Standardized best linear unbiased predictors of *C*. *sativa* families within populations (gray bars), for each treatment separately, for total dry weight, bud set and survival. *TDW* = total dry weight; *BS* = bud set; *S* = survival; *T1* = control treatment; *T2* = drought-stress treatment. Lines in black indicate the families that contribute significantly (*P* < 0.05) to the population variance.
(TIF)

S1 Table Pairwise *F*_*ST*_ estimates and their associated significance levels for the annual growth rhythm experiment. (DOCX)

S2 TableAdditive genetic variances (${\hat{V}}_{A}$), individual heritability estimates (${\hat{h}}_{i}^{2}$) and their standard errors, and additive genetic coefficients of variation (${\hat{CV}}_{A}\%$) for the open pollinated families from the nine C. sativa populations assessed in the annual growth rhythm experiment in 2008.(DOCX)

S3 Table Individual estimated phenotypic correlations (under diagonal) and their significance levels, and additive genetic correlations (above diagonal) and their approximate standard errors (in parentheses), between the traits measured during the 3 years of the annual growth rhythm experiment. (DOCX)

S4 Table Individual estimated phenotypic correlations (under diagonal, *N* = 2,240) between traits and their significance for the combined data of well-watered and periodic drought-stress treatments, and family mean correlations between both treatments (in the diagonal and underlined, *N* = 100). (DOCX)

S1 Dataset Ancestry, molecular and quantitative data for annual growth and drought experiments. The dataset also includes temperature, relative humidity and water deficit records of both treatments of the drought experiment.
(RAR)
