# Supplementary figures and images for: Mediterranean and Northern Iberian gene pools of wild Castanea sativa Mill. are two differentiated ecotypes originated under natural divergent selection
Source: PLoS One. 2019 Feb 12;14(2):e0211315. doi: 10.1371/journal.pone.0211315 (PMC6372156; doi:10.1371/journal.pone.0211315)

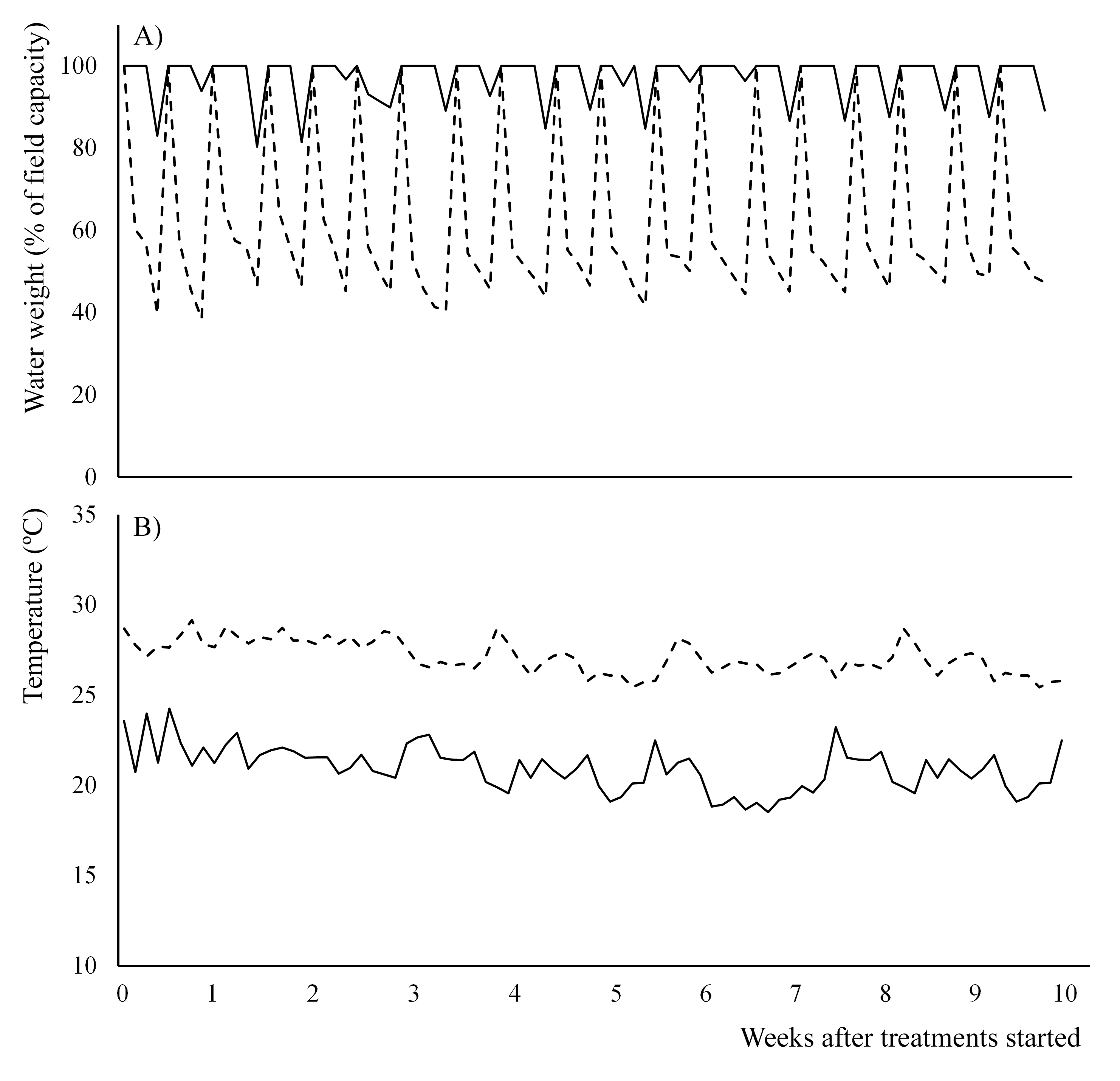

Supplement: S1 Fig — Evolution of the mean water weight (A) and the daily average temperatures (B) for the well-watered (solid line) and the periodic drought-stress (broken line) treatments. Temperature was recorded by eight temperature and humidity sensors that were located at different locations in the two growth chambers. (TIF) [file pone.0211315.s001.tif]

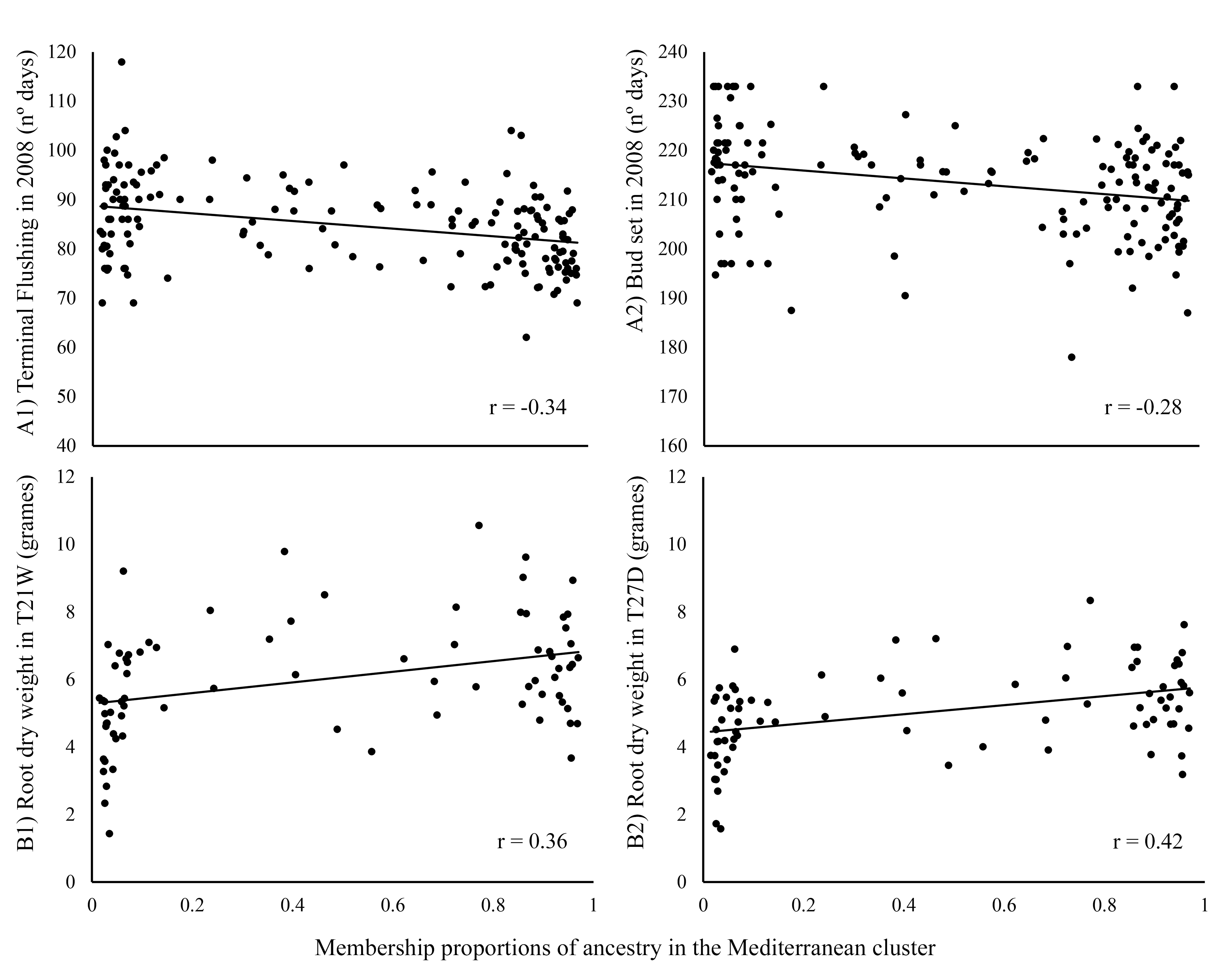

Supplement: S2 Fig — Correlation between the ancestry in the Mediterranean gene pool of the individuals from each population with the means of their corresponding progenies for the number of days to terminal flushing (A1) and to bud set (A2) in 2008 (N = 192) evaluated in the annual growth rhythm experiment, and with the progeny means of root dry weight measured in the well-watered treatment (B1) and in the periodic drought-stress treatment (B2) (N = 90). All of the correlation coefficients were significant at P < 0.001, except for that of B1 (P < 0.01). (TIF) [file pone.0211315.s002.tif]

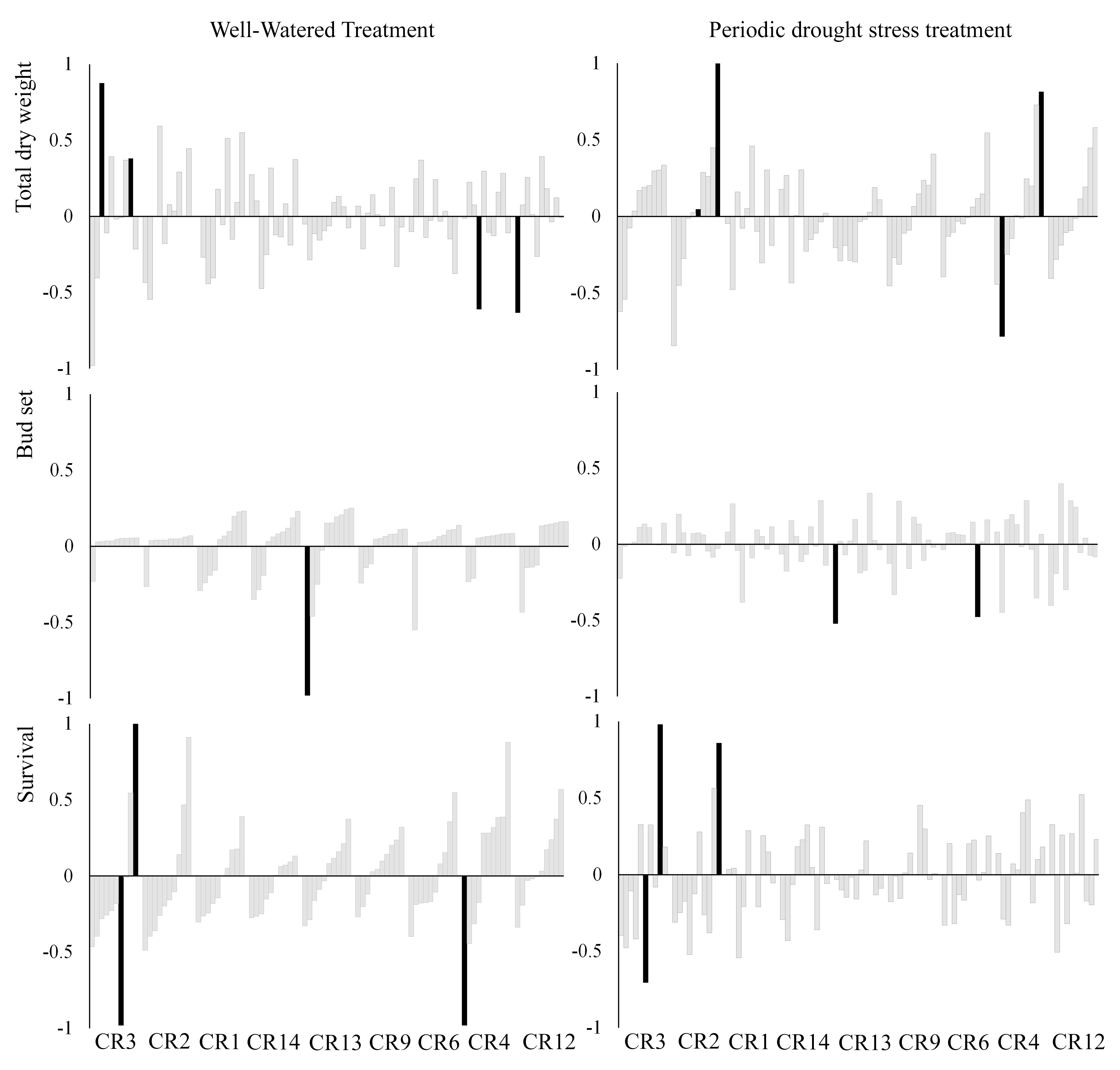

Supplement: S3 Fig — TDW = total dry weight; BS = bud set; S = survival; T1 = control treatment; T2 = drought-stress treatment. Lines in black indicate the families that contribute significantly (P < 0.05) to the population variance. (TIF) [file pone.0211315.s003.tif]
