# Supplementary material for: Mediterranean and Northern Iberian gene pools of wild Castanea sativa Mill. are two differentiated ecotypes originated under natural divergent selection
Source: PLoS One. 2019 Feb 12;14(2):e0211315. doi: 10.1371/journal.pone.0211315 (PMC6372156; doi:10.1371/journal.pone.0211315)
Supplement: S1 Table — (DOCX) [file pone.0211315.s004.docx]

**S1 Table.** **Pairwise *F_ST_* estimates and their associated significance levels for the annual growth rhythm experiment.**

|  | CR1 | CR2 | CR3 | CR4 | CR6 | CR9 | CR12 | CR13 |
| --- | --- | --- | --- | --- | --- | --- | --- | --- |
|  | El Tiemblo | Hervás | Ronda | Maniños | San Cibrán | Nandiello | Eume | Catasós |
| CR1 |  |  |  |  |  |  |  |  |
| CR2 | 0.049** |  |  |  |  |  |  |  |
| CR3 | 0.163*** | 0.164*** |  |  |  |  |  |  |
| CR4 | 0.171*** | 0.175*** | 0.227*** |  |  |  |  |  |
| CR6 | 0.209*** | 0.219*** | 0.263*** | 0.21*** |  |  |  |  |
| CR9 | 0.14*** | 0.161*** | 0.205*** | 0.086*** | 0.18*** |  |  |  |
| CR12 | 0.137*** | 0.129*** | 0.188*** | 0.029* | 0.132*** | 0.112*** |  |  |
| CR13 | 0.092*** | 0.132*** | 0.148*** | 0.133*** | 0.113*** | 0.101*** | 0.097*** |  |
| CR14 | 0.131*** | 0.145*** | 0.202*** | 0.168*** | 0.22*** | 0.184*** | 0.166*** | 0.132*** |

Significance levels: *** *p* < 0.001; ** *p* < 0.01; * *p* < 0.05
