# Supplementary material for: Mediterranean and Northern Iberian gene pools of wild Castanea sativa Mill. are two differentiated ecotypes originated under natural divergent selection
Source: PLoS One. 2019 Feb 12;14(2):e0211315. doi: 10.1371/journal.pone.0211315 (PMC6372156; doi:10.1371/journal.pone.0211315)
Supplement: S2 Table — (DOCX) [file pone.0211315.s005.docx]

**S2 Table.** **Additive genetic variances (**${\hat{\boldsymbol{V}}}_{\boldsymbol{A}}$**), individual heritability estimates (**${\hat{\boldsymbol{h}}}_{\boldsymbol{i}}^{\boldsymbol{2}}$**) and their standard errors, and additive genetic coefficients of variation (**${\hat{\boldsymbol{CV}}}_{\boldsymbol{A}}\boldsymbol{\%}$**) for the open pollinated families from the nine *C. sativa* populations assessed in the annual growth rhythm experiment in 2008.**

|  |  | CR1  El Tiemblo | CR2  Hervás | CR3  Ronda | CR4  Maniños | CR6  San Cibrán | CR9  Nandiello | CR12  Eume | CR13  Catasós | CR14  Mercurín |
| --- | --- | --- | --- | --- | --- | --- | --- | --- | --- | --- |
| TF08 | $\hat{V}_{A}$ | 0.96 | 2.22 | 2.25 | 1.56 | 0.57 | 0.24 | 0.31 | 0.45 | 0.81 |
|  | $\hat{h}_{i}^{2}$ | 0.24 ± 0.11 | 0.47 ± 0.15 | 0.47 ± 0.12 | 0.36 ± 0.18 | 0.16 ± 0.15 | 0.07 ± 0.06 | 0.09 ± 0.31 | 0.13 ± 0.03 | 0.21 ± 0.05 |
|  | $\hat{CV}_{A}$ | na | na | na | na | na | na | na | --- | na |
| LF08 | $\hat{V}_{A}$ | 1.5 | 1.5 | 1.8 | 0.75 | 0.37 | --- | 0.78 | 0.18 | 1.26 |
|  | $\hat{h}_{i}^{2}$ | 0.35 ± 0.09 | 0.35 ± 0.16 | 0.4 ± 0.38 | 0.2 ± 0.45 | 0.1 ± 0.18 | --- | 0.20 ± 0.45 | 0.05 ± 0.03 | 0.3 ± 0.11 |
|  | $\hat{CV}_{A}$ | na | na | na | na | na | --- | na | na | na |
| BS08 | $\hat{V}_{A}$ | 0.27 | 2.52 | --- | 0.99 | --- | 0.54 | --- | 1.26 | 0.48 |
|  | $\hat{h}_{i}^{2}$ | 0.08 ± 0.06 | 0.52 ± 0.07 | --- | 0.25 ± 0.17 | --- | 0.15 ± 0.06 | --- | 0.31 ± 0.22 | 0.13 ± 0.09 |
|  | $\hat{CV}_{A}$ | na | na | --- | na | --- | na | --- | na | na |
| H08 | $\hat{V}_{A}$ | 8.52 | 45.3 | --- | --- | 22.44 | 2.71 | --- | --- | --- |
|  | $\hat{h}_{i}^{2}$ | 0.09 ± 0.09 | 0.44 ± 0.15 | --- | --- | 0.22 ± 0.08 | 0.16 ± 0.1 | --- | --- | --- |
|  | $\hat{CV}_{A}$ | 10.54 | 56.04 | --- | --- | 27.6 | 16.77 | --- | --- | --- |
| RDC08 | $\hat{V}_{A}$ | 0.3 | 1.42 | 0.58 | --- | 0.94 | 0.63 | 0.34 | 0.72 | 0.61 |
|  | $\hat{h}_{i}^{2}$ | 0.14 ± 0.09 | 0.51 ± 0.04 | 0.31 ± 0.1 | --- | 0.4 ± 0.25 | 0.07 ± 0.02 | 0.14 ± 0.13 | 0.04 ± 0.05 | 0.26 ± 0.16 |
|  | $\hat{CV}_{A}$ | 20.12 | 94.42 | 38.21 | --- | 62.15 | 45.82 | 22.71 | 53.25 | 40.64 |
| S08 | $\hat{V}_{A}$ | 0.9 | 1.06 | 0.93 | 1.57 | 0.82 | --- | 1.5 | --- | 0.54 |
|  | $\hat{h}_{i}^{2}$ | 0.25 ± 0.07 | 0.24 ± 0.12 | 0.26 ± 0.09 | 0.41 ± 0.11 | 0.2 ± 0.31 | --- | 0.4 ± 0.37 | --- | 0.16 ± 0.09 |
|  | $\hat{CV}_{A}$ | na | na | na | na | na | --- | na | --- | na |

*TF*, terminal flushing; *LF*, lateral flushing; *BS*, bud set; *H*, height; *RDC*, root collar diameter; *S*, survival. Traits measured in 2008

na, not applicable

Data for straightness and apical dominance were not shown because there were no significant differences between families for most of the populations.
