# Supplementary material for: Mediterranean and Northern Iberian gene pools of wild Castanea sativa Mill. are two differentiated ecotypes originated under natural divergent selection
Source: PLoS One. 2019 Feb 12;14(2):e0211315. doi: 10.1371/journal.pone.0211315 (PMC6372156; doi:10.1371/journal.pone.0211315)
Supplement: S3 Table — (DOCX) [file pone.0211315.s006.docx]

**S3 Table**. **Individual estimated phenotypic correlations (under diagonal) and their significance levels, and additive genetic correlations (above diagonal) and their approximate standard errors (in parentheses), between the traits measured during the 3 years of the annual growth rhythm experiment.**

|  | TF07 | TF08 | LF07 | LF08 | BS07 | BS08 | H06 | H07 | H08 | RCD07 | RCD08 | AD08 | S08 |
| --- | --- | --- | --- | --- | --- | --- | --- | --- | --- | --- | --- | --- | --- |
| TF07 | 1 | 0.98  (0.01) | 1.04  (-0.03) | 0.89 (0.07) | 0.31  (0.26) | ---^a^ | 0.39  (0.23) | 0.5  (0.17) | 0.66  (0.19) | --- | --- | --- | --- |
| TF08 | 0.45*** | 1 | 0.84  (0.12) | 0.94  (0.04) | --- | 0.45  (0.23) | --- | --- | 0.42  (0.19) | --- | --- | --- | --- |
| LF07 | 0.72*** | 0.42*** | 1 | 0.77  (0.15) | 0.41  (0.25) | --- | 0.34  (0.25) | 0.47  (0.21) | 0.64  (0.21) | --- | --- | --- | --- |
| LF08 | 0.45*** | 0.89*** | 0.44*** | 1 | --- | 0.46  (0.23) | --- | --- | 0.38  (0.22) | --- | --- | --- | --- |
| BS07 | 0.17*** | ns | 0.2*** | ns | 1 | 0.51  (0.14) | --- | --- | --- | --- | --- | --- | --- |
| BS08 | 0.08** | 0.11*** | 0.13*** | 0.15*** | 0.32*** | 1 | --- | --- | --- | --- | --- | --- | --- |
| H06 | 0.19*** | 0.17*** | 0.12*** | 0.14*** | 0.14*** | 0.23*** | 1 | 0.94  (0.04) | 0.83  (0.11) | 0.68  (0.18) | 0.46  (0.26) | --- | 0.38  (0.33) |
| H07 | 0.31*** | 0.39*** | 0.27*** | 0.28*** | 0.09** | 0.13*** | 0.6*** | 1 | 0.93  (0.04) | 0.92  (0.04) | 0.56  (0.21) | --- | 0.49  (0.29) |
| H08 | 0.44*** | 0.48*** | 0.33*** | 0.36*** | ns | ns | 0.45*** | 0.77*** | 1 | 0.70  (0.14) | 0.9  (0.07) | -0.4  (0.2) | --- |
| RCD07 | 0.25*** | 0.21*** | 0.22*** | 0.19*** | 0.17*** | 0.28*** | 0.56*** | 0.69*** | 0.44*** | 1 | 0.81  (0.11) | --- | 0.41  (0.32) |
| RCD08 | 0.17*** | 0.33*** | 0.16*** | 0.24*** | 0.13*** | 0.14*** | 0.39*** | 0.5*** | 0.55*** | 0.59*** | 1 | --- | --- |
| AD08 | ns | -0.16*** | ns | -0.15*** | -0.21*** | -0.18*** | --- | -0.08* | -0.31*** | --- | --- | 1 | --- |
| S08 | ns | ns | ns | ns | 0.1** | 0.11*** | 0.1*** | ns | ns | ns | ns | ns | 1 |

*TF*, terminal flushing; *LF*, lateral flushing; *BS*, bud set; *H*, height; *RDC*, root collar diameter; *STR*, straightness; *AD*, apical dominance; *S*, survival; *06*, year 2006; *07*, year 2007; *08*, year 2008

^a^Values that were dismissed because of their high standard error (standard error greater than the estimate)

Significance levels: *** *p* < 0.001; ** *p* < 0.01; * *p* < 0.05; *ns*, not significant
