# Supplementary material for: Mediterranean and Northern Iberian gene pools of wild Castanea sativa Mill. are two differentiated ecotypes originated under natural divergent selection
Source: PLoS One. 2019 Feb 12;14(2):e0211315. doi: 10.1371/journal.pone.0211315 (PMC6372156; doi:10.1371/journal.pone.0211315)
Supplement: S4 Table — (DOCX) [file pone.0211315.s007.docx]

**S4 Table. Individual estimated phenotypic correlations (under diagonal, *N* = 2,240) between traits and their significance for the combined data of well-watered and periodic drought-stress treatments, and family mean correlations between both treatments (in the diagonal and underlined, *N* = 100).**

|  | SDW | RDW | LDW | TDW | RCD | DEF | ABS | NSB | H | BS | DA | S |
| --- | --- | --- | --- | --- | --- | --- | --- | --- | --- | --- | --- | --- |
| SDW | 0.65*** |  |  |  |  |  |  |  |  |  |  |  |
| RDW | 0.85*** | 0.57*** |  |  |  |  |  |  |  |  |  |  |
| LDW | 0.77*** | 0.79*** | 0.27** |  |  |  |  |  |  |  |  |  |
| TDW | 0.95*** | 0.96*** | 0.89*** | 0.55*** |  |  |  |  |  |  |  |  |
| RCD | 0.48*** | 0.43*** | 0.34*** | 0.46*** | 0.27* |  |  |  |  |  |  |  |
| DEF | -0.15*** | -0.25*** | -0.39*** | -0.26*** | -0.11*** | *ns* |  |  |  |  |  |  |
| ABS | *ns* | -0.09** | -0.22*** | -0.09** | 0.06** | 0.49*** | *ns* |  |  |  |  |  |
| NSB | -0.05* | -0.08** | -0.11*** | -0.08** | -0.06** | 0.18*** | 0.06* | 0.25* |  |  |  |  |
| H | 0.76*** | 0.54*** | 0.53*** | 0.65*** | 0.5*** | 0.07** | 0.16*** | *ns* | 0.73*** |  |  |  |
| BS | 0.38*** | 0.17*** | 0.29*** | 0.29*** | 0.18*** | *ns* | 0.09** | *ns* | 0.58*** | 0.48*** |  |  |
| DA | -0.35*** | -0.27*** | -0.33*** | -0.34*** | -0.19*** | 0.18*** | 0.02^ns^ | 0.05* | -0.35*** | -0.51*** | 0.31*** |  |
| S | 0.18*** | 0.23*** | 0.08** | 0.23*** | *ns* | *ns* | *ns* | *ns* | *ns* | -0.05* | *ns* | 0.48*** |

*SDW*, stem dry weight; *RDW*, root dry weight; *LWD*, leaf dry weight; *TWD*, total dry weight; *RDC*, root collar diameter; *DEF*, defoliation; *NSB* number of secondary branches; *H*, height; *BS*, bud set; *DA*, dry apex; *S*, survival

Significance levels: *** *p* < 0.001; ** *p* < 0.01; * *p* < 0.05; *ns*, not significant
